# Induction of ferroptosis in prostate cancer by CCDC7_19-13_ via TRIM21-mediated ubiquitination of SLC7A11

**DOI:** 10.1038/s41418-025-01580-x

**Published:** 2025-09-22

**Authors:** Bisheng Cheng, Qiong Wang, Zean Li, Tianlong Luo, JunJia Xie, Sandeep Singh, Yong Luo, Xu Gao, Hui Li, Zongwei Wang, Peng Wu, Hai Huang

**Affiliations:** 1 Department of Urology, Nanfang Hospital, Southern Medical University, Guangzhou, China; 2 Department of Urology, Sun Yat-sen Memorial Hospital, Sun Yat-sen University, Guangzhou, China; 3 Department of Surgery, Division of Urology, Beth Israel Deaconess Medical Center, Harvard Medical School Boston, Boston, MA USA; 4 Department of Pathology, School of Medicine, University of Virginia, Charlottesville, VA USA; 5 Department of Urology, Changhai Hospital, Second Military Medical University, Shanghai, China; 6 Guangdong Provincial Clinical Research Center for Urological Diseases, Sun Yat-Sen Memorial Hospital, Sun Yat-Sen University, Guangzhou, China; 7 Department of Urology, The Sixth Affiliated Hospital of Guangzhou Medical University, Qingyuan People’s Hospital, Qingyuan, Guangdong China; 8 Guangdong Provincial Key Laboratory of Malignant Tumor Epigenetics and Gene Regulation, Sun Yat-Sen Memorial Hospital, Sun Yat-Sen University, Guangzhou, China

**Keywords:** Cancer, Tumour-suppressor proteins

## Abstract

Prostate cancer is one of the most prevalent malignancies in men, with increasing incidence and mortality largely attributed to treatment resistance and metastasis. The effectiveness of current therapies for advanced cases is hindered by intricate genetic and microenvironmental factors, emphasizing the urgent need for novel therapeutic targets. Chimeric RNAs have emerged as promising biomarkers in cancer research, among which CCDC7_19-13_, a circular chimeric RNA, is frequently identified in prostate cancer. Our study reveals that CCDC7_19-13_ expression is markedly reduced in advanced and recurrent prostate cancer, where its low levels serve as an independent predictor of poor prognosis. Functional experiments demonstrate that CCDC7_19-13_ overexpression inhibits cell proliferation, induces apoptosis, and suppresses tumor growth in vivo, whereas its knockdown reverses these effects. Mechanistically, CCDC7_19-13_ encodes a novel protein, CCDC7_241aa_, which triggers ferroptosis by interacting with SLC7A11 and facilitating its TRIM21-mediated ubiquitination and degradation. Notably, treatment with recombinant CCDC7_241aa_ effectively suppresses tumor growth in patient-derived xenograft models without toxicity and enhances the efficacy of docetaxel and enzalutamide in vitro. These findings establish CCDC7_19-13_ as a significant prognostic marker and potential therapeutic target in prostate cancer, with the recombinant CCDC7_241aa_ protein offering promise for combination therapies in advanced cases.

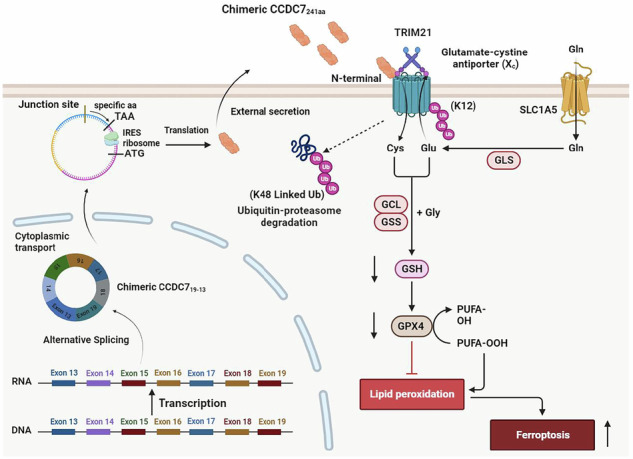

## Introduction

Prostate cancer is one of the most prevalent malignancies in male, posing a significant global health challenge [1, 2]. Despite advancements in diagnostic and therapeutic strategies, the disease’s complexity and heterogeneity continue to complicate its management [3–5]. The development of resistance to conventional therapies and the recurrence of aggressive forms of the disease underscore the urgent need for novel therapeutic strategies. High-throughput sequencing technologies have identified chimeric RNAs as crucial players in cancer biology, including prostate cancer [6–9]. These hybrid transcripts, generated through gene fusions, trans-splicing, or read-through events, can encode functional proteins or act as non-coding RNAs, influencing tumor progression via diverse mechanisms [10–12]. Increasing evidence suggests that chimeric RNAs hold significant potential as diagnostic, prognostic, and therapeutic targets [13, 14]. Given the genetic alterations frequently observed in prostate cancer, investigating these chimeric proteins is of particular importance.

Among the mechanisms governing cancer progression, ferroptosis—a regulated form of cell death driven by iron accumulation and lipid peroxidation—has garnered considerable attention [15–17]. Unlike apoptosis, ferroptosis is primarily triggered by excessive lipid peroxides, leading to cellular damage and death [18–20]. Notably, this pathway presents a unique vulnerability in cancer cells, particularly those resistant to conventional therapies, making it a promising therapeutic target.

In this study, we demonstrate that CCDC7_19-13_, a chimeric RNA, is significantly downregulated in high-grade, recurrent, and metastatic prostate cancer, with its low expression serving as an independent predictor of poor prognosis. CCDC7_19-13_ encodes the novel protein CCDC7_241aa_, which induces ferroptosis by interacting with SLC7A11 and facilitating its TRIM21-mediated ubiquitination and degradation. This process disrupts redox homeostasis, thereby triggering ferroptosis and inhibiting tumor progression [21–23]. Functional studies reveal that overexpression of CCDC7_19-13_ suppresses cell proliferation, enhances ferroptosis, and reduces tumor growth, while its knockdown reverses these effects. Moreover, in patient-derived xenograft (PDX) models, recombinant CCDC7_241aa_ effectively inhibits tumor growth without toxicity and synergizes with docetaxel and enzalutamide in vitro. These findings establish CCDC7_19-13_ as a crucial prognostic biomarker and therapeutic target in advanced prostate cancer. By modulating the TRIM21-SLC7A11 axis and inducing ferroptosis, CCDC7_19-13_ provides a promising strategy to combat treatment resistance and prevent disease progression.

## Materials and methods

### Bioinformatics

We analyzed 134 paired prostate cancer and adjacent non-tumor tissue samples collected from Shanghai Changhai Hospital between 2014 and 2019 [24]. Chimeric RNAs were identified using EricScript (v0.5.5b) with the hg38 reference genome and default parameters. Chimeras with EricScores below 0.6 were excluded to improve specificity. To ensure cancer-specificity, we cross-referenced the identified chimeras with those found in healthy tissues from previous studies. Gene Ontology (GO) analysis of the parental genes was performed using GOrilla (http://cbl-gorilla.cs.technion.ac.il/) with the hg38 genomic background. Read counts for target chimeras were quantified across TCGA and other datasets using Agrep (https://www.tgries.de/agrep/). This workflow ensures a robust identification of chimeric RNAs relevant to prostate cancer. All data, including raw data, mutation calls, and clinical information, have been deposited to the Genome Sequence Archive for Human (http://bigd.big.ac.cn/gsa-human/) at the BIG Data Center, Beijing Institute of Genomics, Chinese Academy of Sciences, under the accession number PRJCA001124.

### Clinical samples

Tissue samples, including both tumor and adjacent normal tissues, were collected from 202 prostate cancer (PCa) patients at Sun Yat-sen Memorial Hospital, Southern Medical University Nanfang Hospital and Sun Yat-sen University Cancer Center [5, 25]. These samples were analyzed to evaluate CCDC7₁₉₋₁₃ expression and its clinical significance. The study population was divided into two cohorts: Cohort 1 consisted of 78 prostate cancer specimens, each paired with a corresponding adjacent normal tissue sample from Sun Yat-sen Memorial Hospital. Meanwhile, Cohort 2 included 124 prostate cancer tissues and 65 matched normal tissue samples obtained from Sun Yat-sen University Cancer Center. All collected specimens were immediately preserved by snap-freezing in liquid nitrogen at −80 °C to maintain sample integrity for subsequent analysis. The research protocol was approved by the Ethics Committee of Sun Yat-sen University (Approval No. SYSKY-2023-925-01), ensuring compliance with ethical guidelines. Additionally, written informed consent was secured from all participants prior to sample collection.

### Cell lines and cell culture

Cell culture experiments were performed following previously established protocols [26]. Human prostate cancer (PCa) cell lines, including LNCaP (RRID:CVCL_1379), IE8 (RRID:CVCL_2L33), PC3 (RRID:CVCL_0035), C4-2 (RRID:CVCL_4783), DU145 (RRID:CVCL_0105), and 22RV1 (RRID:CVCL_1045), were utilized to investigate the cellular mechanisms underlying prostate malignancies. All cell lines were authenticated using short tandem repeat (STR) analysis before experimental use, and were confirmed to be free of mycoplasma contamination by PCR-based testing. LNCaP, IE8, PC3, C4-2, and 22RV1 cell lines were obtained from the American Type Culture Collection (ATCC) between 2019 and 2021. DU145 and HEK-293T (RRID:CVCL_0063) cells were purchased from the Type Culture Collection of the Chinese Academy of Sciences (Shanghai) in 2020. The RWPE-1 cell line (RRID:CVCL_3791), derived from normal prostate epithelium, was obtained from the Cell Center of the Shanghai Institutes for Biological Sciences in 2020 and served as a benign prostatic epithelial control. RWPE-1 cells were maintained in Keratinocyte Serum-Free Medium (K-SFM, Gibco) supplemented with 0.05 mg/mL bovine pituitary extract (BPE), 5 ng/mL epidermal growth factor (EGF), and 1% penicillin-streptomycin (P/S) (Gibco, USA). DU145 and HEK-293T cells were cultured in Dulbecco’s Modified Eagle Medium (DMEM, Gibco), while all other PCa cell lines were maintained in RPMI 1640 medium (Gibco). Both RPMI 1640 and DMEM were supplemented with 10% fetal bovine serum (FBS, Invitrogen) and 1% penicillin-streptomycin (Gibco) to support optimal cell growth and viability. All cell cultures were maintained at 37 °C in a humidified incubator containing 5% CO.

### RNA extraction, gDNA extraction, and quantitative real-time PCR analysis

The procedures for RNA extraction and quantitative real-time PCR (qRT-PCR) followed previously established protocols [27]. Total RNA was extracted from tissues and cultured cells using TRIzol reagent (Invitrogen, Grand Island, NY, USA), in accordance with the manufacturer’s guidelines. Subsequently, 1 μg of RNA was reverse-transcribed using the HiScript II One Step qRT-PCR Kit (Vazyme, China; cat. no. Q221-01) in a 20 μL reaction system. For genomic DNA (gDNA) extraction, tissue samples were processed using the PureLink Genomic DNA Mini Kit (Thermo Fisher Scientific, K182001) according to the provided instructions. Real-time PCR (qPCR) was conducted on an ABI QuantStudio DX system (Applied Biosystems, USA) employing SYBR Green as the fluorescent dye for DNA-specific detection. Human GAPDH was selected as the internal housekeeping gene for normalization. Relative gene expression levels were determined using the comparative CT method (ΔΔCT), with fold enrichment calculated as 2^^[ΔCT(sample) - ΔCT(calibrator)]^. A comprehensive list of all primers used in this study can be found in Supplementary Table 8.

### Fluorescence in situ hybridization (FISH)

Fluorescence in situ hybridization (FISH) was employed to analyze CCDC7_19-13_ expression in prostate cancer cells and tissues using a FITC-labeled probe (RiboBio, Guangzhou, China). Cells were washed with PBS, fixed with 4% formaldehyde (Sigma-Aldrich), and permeabilized using 0.1% Triton X-100 in PBS before being mounted onto coverslips and incubated with CCDC7_19-13_-targeting probes. Hybridization was performed in a specialized hybridization solution (Boster, probe dilution 1:1000) at 52 °C for 16 h under humidity-controlled conditions. Following hybridization, an extensive washing process was carried out, consisting of a 25% deionized formamide and 2× SSC wash at 52 °C for 30 min, followed by a final rinse in 2× SSC. After the washing steps, cells were incubated at 4 °C for 12 h with a fluorescein-conjugated Digoxin antibody (Roche, cat. 11207741910, dilution 1:100). Finally, DAPI counterstaining was performed to visualize cell nuclei.

### Plasmids and cell transfection

The CCDC7₁₉₋₁₃ overexpression(OE) vector (pCDH-CMV-MCS-EF1) and its corresponding control plasmid were procured from IGE Biotechnology Company (Guangzhou, China). Additionally, the CCDC7₁₉₋₁₃×FLAG coding sequence was incorporated into the same vector. Transient transfection, lentiviral packaging, and infection procedures were carried out following established protocols. For transient transfection, plasmids were mixed with X-tremeGENE (Invitrogen) and incubated at 25 °C for 20 min before being introduced into target cells, which were subsequently cultured for 24–48 h. For lentivirus production, HEK-293T cells were co-transfected with psPAX2 and PMD2.G (IGE) using X-tremeGENE. After 48 h, the resulting lentiviral particles were harvested, filtered, and concentrated. Target cells were then infected in the presence of polybrene (IGE) and subjected to puromycin selection (Table 6).

### RNase R treatment

RNase R treatment was performed according to established protocols [12]. In the control group, 2 μg of total RNA was combined with 0.2 μL of RNase-free water and 0.6 μL of 10× RNase R Reaction Buffer (Epicentre Technologies). For the RNase R-treated group, 0.2 μL of RNase R (20 U/μL) was introduced into the same reaction mixture to evaluate its impact. Samples were then incubated at 37 °C for 30 min to facilitate enzymatic activity before proceeding with reverse transcription. GAPDH served as an internal control in the untreated group, providing a stable reference for assessing relative changes and ensuring experimental consistency.

### Subcellular isolation of RNA and protein

To separate RNA and protein from nuclear and cytoplasmic fractions, specialized subcellular fractionation kits were employed. RNA was isolated using the RNA Subcellular Isolation Kit (Active Motif), while protein was extracted with the Nuclear and Cytoplasmic Protein Extraction Kit (Beyotime). All procedures adhered strictly to manufacturer protocols to ensure precision and reproducibility. Compliance with standardized methodologies was critical for maintaining sample integrity and establishing a robust foundation for subsequent analyses.

### Cell proliferation assays

Cell proliferation was assessed using the methyl thiazolyl tetrazolium (MTT) assay [28]. Cells were seeded into 96-well plates and incubated with MTT labeling reagent for 2 h. Following incubation, the formazan crystals formed were dissolved using dimethyl sulfoxide (DMSO), and the absorbance at 570 nm was measured to determine cell viability. For the colony formation assay, 500 prostate cancer (PCa) cells were plated into six-well plates and maintained under standard conditions for 10 days. Colonies were subsequently fixed with 4% paraformaldehyde and stained with crystal violet for 20 min. The stained colonies were then visualized and analyzed using Quantity One 1-D Analysis Software. All measurements were normalized to Day 1 values and expressed as mean ± standard deviation (SD). Each experiment was performed in triplicate to ensure data reliability and reproducibility.

### Flow cytometry analysis of ROS level

PC3 and DU145 cells were seeded in 6-well plates at 5 × 10⁵ cells/well and cultured overnight. Cells were then treated with DCFH-DA (10 μM) for 20 min, followed by two PBS washes. Finally, they were harvested and analyzed via flow cytometry using a 488 nm excitation laser.

### Cellular iron staining

Cells were rinsed twice with PBS, followed by staining with 10 nM Phen Green™ SK at 37 °C for 15 min. After staining, the cells were centrifuged, resuspended in PBS, and their fluorescence intensity was measured using a BD Aka Fortessa X30 system. A higher fluorescence intensity corresponded to lower intracellular iron levels.

### Lipid peroxidation and GSH analysis

Tumor cells were seeded into six-well plates, exposed to the specified treatments, and incubated for 48 h. For BODIPY-C11 staining, PC3 and DU145 cells were treated with 2 μM BODIPY-C11 at 37 °C for 30 min, then collected through trypsinization(DOJINDO, L267). The cells were then washed twice with PBS and immediately analyzed using a flow cytometer (Beckman PLUS). FACS data were processed using FlowJo 7.6. Intracellular GSH levels were quantified with a GSH detection kit (Beyotime, S0053), following the manufacturer’s protocol. All results were normalized to the corresponding protein concentrations.

### Western blotting and analysis

Western blotting was performed following previously established protocols [29]. Total protein extraction was carried out using WB/IP lysis buffer (Beyotime), supplemented with protease and phosphatase inhibitors (Selleck) to maintain protein stability. Equal amounts (30 μg) of protein were resolved by 10% SDS-PAGE and subsequently transferred onto polyvinylidene fluoride (PVDF) membranes (Merck Millipore). Membranes were incubated overnight at 4 °C with primary antibodies (referenced in Table 7), followed by exposure to a secondary antibody (Promega, CST) at a 1:10,000 dilution for 1 h at ambient temperature. Protein bands were detected using a chemiluminescent HRP substrate (Merck Millipore). Adhering strictly to the experimental protocol ensured high reproducibility and data accuracy, reinforcing the scientific validity of the study.

### Co‐immunoprecipitation (Co‐IP) and mass spectrometry (MS) analysis

Co‐immunoprecipitation (Co‐IP) was conducted following previously established protocols [30] to examine the interaction between CCDC7_241aa_ and SLC7A11 in wild-type PC3 and DU145 cells. Nuclear extracts were incubated overnight at 4 °C with anti-Flag, anti-SLC7A11, or control IgG antibodies (Supplementary Table S2) to facilitate binding. Subsequently, A/G magnetic beads were added, and samples were incubated for an additional 2 h at 25 °C to enhance complex formation. Immunoreactive proteins were detected via Western blotting. Protein bands near 42 kDa were excised, cut into 1 mm³ fragments, and processed for peptide extraction and drying before undergoing LC–MS/MS analysis. The mass spectrometry (MS) study was performed at the Bioinformatics and Omics Center of Sun Yat-Sen Memorial Hospital to identify protein interactions. LC–MS/MS-based protein identification followed a previously validated approach [31].

### Orthotopic prostate cancer transplantation model

Male 4-week-old male BALB/c mice, obtained from the Experimental Animal Center of Sun Yat-sen University, were used for in vivo experiments. Mice were weighed and anesthetized before surgery. A 1.5 cm midline incision was made in the lower abdomen to expose the prostate and bladder. Under a surgical microscope, a microsyringe was used to inject 1 × 10^6^ cells suspended in 10 µL into the left anterior lobe of the prostate, forming a raised bleb at the injection site. After the injection, the prostate and bladder were carefully repositioned, and the incision was sutured with double-layer, interrupted stitches. Postoperative treatments were administered according to group assignments, starting the day after surgery and continuing until the experimental endpoint. Animals were randomly assigned to experimental groups unless otherwise stated.

### Establishment of prostate cancer patient‐derived xenografts (PDX) models

Prostate cancer PDX models were developed using male NOD-SCID mice, aged between 4 and 5 weeks, supplied by Guangzhou JNO Laboratory Animal Technology and the Experimental Animal Center at Sun Yat-sen University, respectively. These mice were maintained in specific pathogen-free (SPF) conditions. Tumor specimens, sourced from patients with castration-resistant prostate cancer at Sun Yat-sen Memorial Hospital, were processed following surgical removal. The specimens were promptly preserved in sterile DMEM at cold temperatures post-resection. Within a six-hour timeframe, these specimens were sectioned into pieces about 3 mm in diameter and then subcutaneously transplanted onto the dorsum’s right side of NOD-SCID mice, initiating the growth of first-generation (F1) tumors. Approximately eight weeks later, once the F1 tumors reached a size close to 100 mm^3^, a humane end-of-life procedure was carried out through cervical dislocation. The xenografts were then carefully extracted and reimplanted into NOD-SCID mice, adhering to the initial protocol. As soon as the tumors became externally noticeable, achieving around 5 mm in diameter, the mice were segregated into two experimental cohorts, each consisting of five mice with comparable average tumor volumes. These groups were then systematically assigned to undergo treatment with either the experimental recombinant protein CCDC7_241aa_ or a control vehicle, following the previously established protocol.

### Detection of Malondialdehyde (MDA) and 4-Hydroxynonenal (4-HNE)

Lipid peroxidation levels were evaluated using commercially available detection kits: MDA (KTE61683, Abbkine, USA) and 4-HNE (ab238538, Abcam, UK). For the MDA assay, 2 × 10⁶ cells were disrupted in MDA lysis buffer containing 1% butylated hydroxytoluene (BHT) to prevent oxidation. After centrifugation, 200 μL of the supernatant was combined with 600 μL of Developer VII/TBA reagent and heated at 95 °C for 1 h. The mixture was then cooled on ice, and 200 μL was transferred to a 96-well plate for absorbance measurement at 532 nm. For the 4-HNE assay, 1 × 10⁶ cells were lysed in RIPA buffer, and the supernatant was collected post-centrifugation. A 50 μL aliquot of the supernatant was added to a 4-HNE conjugate-coated plate and incubated at room temperature for 10 min. Detection was conducted through sequential incubation with an anti-4-HNE primary antibody, followed by an HRP-conjugated secondary antibody and substrate addition. The absorbance was measured at 450 nm.

### Immunohistochemistry staining

Tumor tissues were aseptically excised, fixed in 4% neutral buffered formalin, and embedded in paraffin. Serial 5-μm-thick sections were stained with hematoxylin and eosin (H&E) for histopathological evaluation. To assess lipid peroxidation, MDA levels were detected. Immunohistochemical staining was carried out following previously established protocols [28, 32]. Images were acquired using a DP21 digital camera.

### Statistical analysis

Statistical tests were chosen based on the distribution and variance characteristics of the data. All statistical analyses were performed using R software (version 4.0.2, R Foundation for Statistical Computing, Vienna, Austria) [33]. Survival analysis was conducted via the Kaplan–Meier method, and log-rank tests were utilized to assess differences in survival curves. The cut-off value for CCDC7₁₉₋₁₃ expression was determined as follows: In the primary cohort, the median value served as the threshold. For other cohorts, the optimal cut-off point was computed using the ‘surv_cutpoint’ function within the ‘survminer’ package (version 0.4.9, R) to optimize p-value minimization in survival analysis. To ensure the reproducibility and consistency of cut-off values across cohorts, rigorous validation was conducted. For group comparisons, a Student’s *t* test was applied for two-group analyses, whereas one-way analysis of variance (ANOVA) was employed for comparisons involving multiple groups (*n* > 2). Statistical significance was defined as *p* < 0.05.

## Results

### Identification and characteristics of CCDC7_19-13_ in PCA

**Fig. 1:**
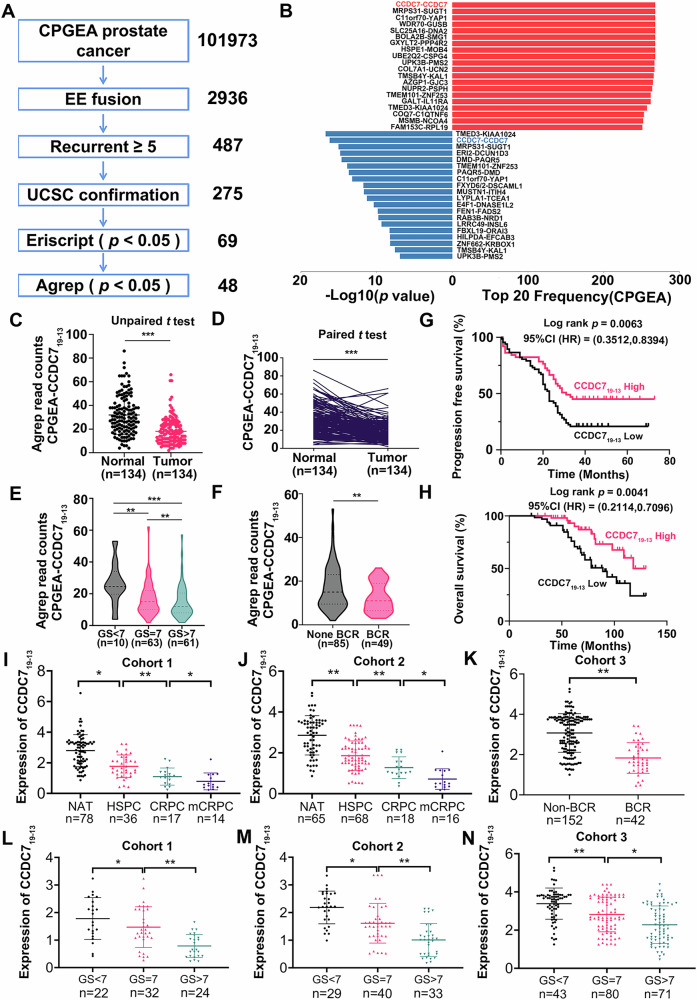
Identification and clinical relevance of chimeric RNA CCDC7_19-13_ in prostate cancer. **A** Workflow for identifying chimeric RNAs in the CPGEA prostate cancer dataset. Starting from 101,973 initial reads, 2936 EE fusions were identified. Filtering for recurrent events (≥5 times) yielded 487 candidates, further confirmed through UCSC (275 candidates), Eriscript (69 candidates, *p* < 0.05), and Agrep analysis (48 candidates, *p* < 0.05). **B** Top 20 chimeric RNAs by frequency in the CPGEA dataset, ranked by -log10(*p*-value). CCDC7_19-13_ is highlighted for its significant differential expression and high frequency. **C** Comparison of CCDC7_19-13_ expression levels in normal (*n* = 134) and tumor (*n* = 134) tissues using an unpaired t-test, showing significantly lower expression in tumor tissues. **D** Paired *t*-test comparison of CCDC7_19-13_ expression in matched normal and tumor tissues (*n* = 134), showing significantly reduced expression in tumor tissues. **E** Violin plot of CCDC7_19-13_ expression stratified by Gleason score (GS): GS ≤ 7 (*n* = 10) and GS > 7 (*n* = 63). **F** Violin plot comparing CCDC7_19-13_ expression in non-biochemically recurrent (Non-BCR, *n* = 85) and biochemically recurrent (BCR, *n* = 49) prostate cancer tissues, showing lower expression in BCR tissues. **G** Kaplan–Meier analysis of progression-free survival in prostate cancer patients with high versus low CCDC7_19-13_ expression. Higher expression correlates with better prognosis (log-rank *p* = 0.0063, HR = 0.3512, CI: 0.3512–0.8394). **H** Kaplan–Meier analysis of overall survival in prostate cancer patients with high versus low CCDC7_19-13_ expression, showing improved survival with higher expression (log-rank *p* = 0.0041, HR = 0.2114, CI: 0.2114–0.7096). **I**, **J** Expression of CCDC7_19-13_ in different cohorts. **I** Cohort 1 stratified by normal adjacent tissue (NAT, *n* = 78), high-risk primary prostate cancer (HSPC, *n* = 36), castration-resistant prostate cancer (CRPC, *n* = 17), and metastatic CRPC (mCRPC, *n* = 14). **J** Cohort 2 stratified by NAT (*n* = 65), HSPC (*n* = 68), CRPC (*n* = 18), and mCRPC (*n* = 16). **K** Expression of CCDC7_19-13_ in Cohort 3, comparing Non-BCR (*n* = 152) and BCR (*n* = 42) prostate cancer tissues. **L**–**N** Expression of CCDC7_19-13_ stratified by Gleason score in different cohorts. **L** Cohort 1: GS ≤ 7 (*n* = 22) and GS > 7 (*n* = 32 and *n* = 24). **M** Cohort 2: GS ≤ 7 (*n* = 29) and GS > 7 (*n* = 40 and *n* = 33). **N** Cohort 3: GS ≤ 7 (*n* = 43) and GS > 7 (*n* = 80 and *n* = 71). The asterisks denote the level of statistical significance as follows: *p < 0.05, **p < 0.01, ***p < 0.001.

To identify key fusion gene events associated with prostate cancer progression and treatment resistance, we analyzed the largest prostate cancer transcriptome dataset from the Chinese Prostate Cancer Genome and Epigenome Atlas (CPGEA) dataset (GSA-Human, PRJCA001124). This analysis identified 2936 gene fusion events in prostate cancer. After filtering out low-frequency fusions (occurrence <5) and misreads, and conducting Approximate Global Regular Expression Print (Agrep) matching analysis, we identified 48 fusion genes significantly differentially expressed between prostate cancer and adjacent normal tissues (Fig. 1A). Among these, the fusion gene CCDC7_19-13_, involving exons of CCDC7, was the most frequent and significantly differentially expressed (Fig. 1B). Further clinical data analysis revealed that CCDC7_19-13_ expression was markedly reduced in prostate cancer tissues and was particularly low in high-grade (Gleason score, GS) and biochemically recurrent (BCR) prostate cancers (Fig. 1C–F, Table 1). Therefore, CCDC7_19-13_ became the focus of our subsequent research.

To validate the expression of CCDC7_19-13_, we employed quantitative PCR and Fluorescence In Situ Hybridization (FISH) in multiple clinical cohorts. Analysis included 78 prostate cancer tissues and paired adjacent normal tissues from our center (Cohort 1) and 102 prostate cancer tissues and 65 adjacent normal tissues from Sun Yat-sen University Cancer Center (Cohort 2). The results confirmed that CCDC7_19-13_ expression was significantly lower in prostate cancer tissues compared to normal adjacent tissues (NAT). Furthermore, CCDC7_19-13_ expression progressively decreased in hormone-sensitive prostate cancer (HSPC), castration-resistant prostate cancer (CRPC), and metastatic CRPC (mCRPC) (Fig. 1I, J, Supplementary Fig. S1A). Using a cohort of prostate cancer patients from Nanfang Hospital, Southern Medical University, (Cohort 3), we found that CCDC7_19-13_ expression was significantly lower in biochemically recurrent (BCR) prostate cancer tissues compared to non-BCR tissues (Fig. 1K). Additionally, CCDC7_19-13_ expression was significantly reduced in high GS prostate cancers compared to low GS prostate cancers across multiple cohorts (Fig. 1L–N). Further Kaplan–Meier analysis demonstrated that high CCDC7_19-13_ expression was associated with improved progression-free survival and overall survival in prostate cancer patients (Fig. 1G, H, Supplementary Fig. S1B–G). These results collectively indicate that CCDC7_19-13_ is significantly underexpressed in high-grade, recurrent, and metastatic prostate cancer tissues, suggesting its potential role as a prognostic marker and therapeutic target. Consistently, multivariate Cox regression analyses in Cohort 1 and 2 confirmed that low CCDC7_19-13_ expression was an independent predictor of poor overall and progression-free survival (Supplementary Tables 2–5).

### CCDC7_19-13_ inhibits prostate cancer cell proliferation and promotes apoptosis

**Fig. 2:**
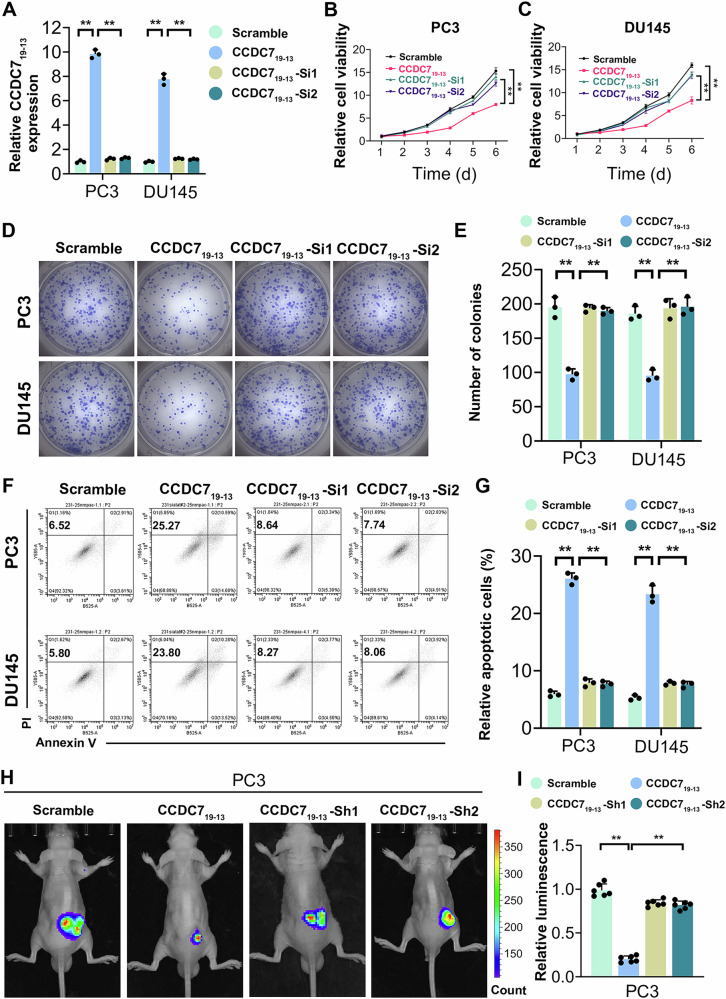
CCDC7_19-13_ inhibits prostate cancer cell proliferation and promotes apoptosis in vitro and suppresses tumor growth in vivo. **A** Relative expression of CCDC7_19-13_ in PC3 and DU145 cells transfected with scramble control, CCDC7_19-13_ overexpression vector, or CCDC7_19-13_ overexpression combined with specific siRNAs. Cell viability of **B** PC3 and **C** DU145 cells with the same transfections. **D** Colony formation assay in PC3 and DU145 cells after the indicated transfections. Colonies were stained with crystal violet. **E** Quantification of colony numbers from (**D**). **F** Flow cytometry scatter plots showing apoptosis in PC3 and DU145 cells after transfections. **G** Quantification of apoptotic cells from (**F**). **H** Bioluminescence images of PC3 xenografts in BALB/c mice injected with cells transfected as indicated. **I** Quantification of bioluminescence intensity from (**H**). Data are presented as mean ± SD. *p < 0.05, **p < 0.01, ***p < 0.001.

To investigate the roles of CCDC7_19-13_ in prostate cancer (PCa), we first evaluated the expression of CCDC7_19-13_ in different PCa cell lines. Based on its moderate endogenous expression in PC3 and DU145 cells, these two cell lines were selected for functional assays. We performed overexpression(OE) of CCDC7_19-13_ using a CCDC7_19-13_ overexpression vector and further knocked down CCDC7_19-13_ expression on the basis of CCDC7_19-13_ overexpression using two specific siRNAs (Si1 and Si2). Cell viability assays revealed that overexpression of CCDC7_19-13_ significantly inhibited cell proliferation in both PC3 and DU145 cells compared to the scramble control. Notably, further knockdown of CCDC7_19-13_ on the basis of its overexpression partially restored cell proliferation, indicating a specific role of CCDC7_19-13_ in suppressing PCa cell growth (Fig. 2A–C). Consistently, colony formation assays showed that overexpression of CCDC7_19-13_ led to a significant reduction in the number of colonies formed, while subsequent knockdown of CCDC7_19-13_ on the overexpression background restored colony-forming ability (Fig. 2D, E). Flow cytometry analysis revealed a significant increase in apoptosis in PC3 and DU145 cells overexpressing CCDC7_19-13_ compared to the scramble control. Moreover, apoptosis was significantly reduced when CCDC7_19-13_ was knocked down on the basis of overexpression (Fig. 2F, G). These results suggest that CCDC7_19-13_ plays a critical role in promoting apoptosis and inhibiting proliferation in PCa cells.

To evaluate the in vivo effects of CCDC7_19-13_, we generated an orthotopic prostate cancer model using BALB/c mice. Bioluminescence imaging showed that tumor growth was significantly reduced in the CCDC7_19-13_ overexpression group compared to the scramble control. However, knockdown of CCDC7_19-13_ in the overexpression group partially restored tumor growth (Fig. 2H). Quantification of bioluminescence intensity confirmed these findings, demonstrating that overexpression of CCDC7_19-13_ significantly suppressed tumor growth in vivo, while its knockdown reversed this effect (Fig. 2I, Supplementary Fig. S3A, B). Kaplan–Meier survival analysis further demonstrated that mice bearing PC3 xenografts with CCDC7_19-13_ overexpression exhibited significantly improved survival compared to controls, with reduced survival observed in the knockdown group (Supplementary Fig. S3C).

These results suggest that CCDC7_19-13_ exerts its biological effects in prostate cancer by inhibiting cell proliferation and promoting apoptosis. The intrinsic reasons for cell proliferation can be attributed to either accelerated cell cycle progression or hindered apoptosis [34, 35]. Further analysis showed that overexpression or knockdown of CCDC7_19-13_ did not significantly affect the cell cycle distribution at the G0/G1, S, or G2/M phases (Supplementary Fig. S2A–C), indicating that CCDC7_19-13_ predominantly influences apoptosis rather than cell cycle progression as the primary mechanism for its anti-proliferative effects.

### Inhibitory effects of the circular structure and protein-coding potential of CCDC7_19−13_ on prostate cancer

**Fig. 3:**
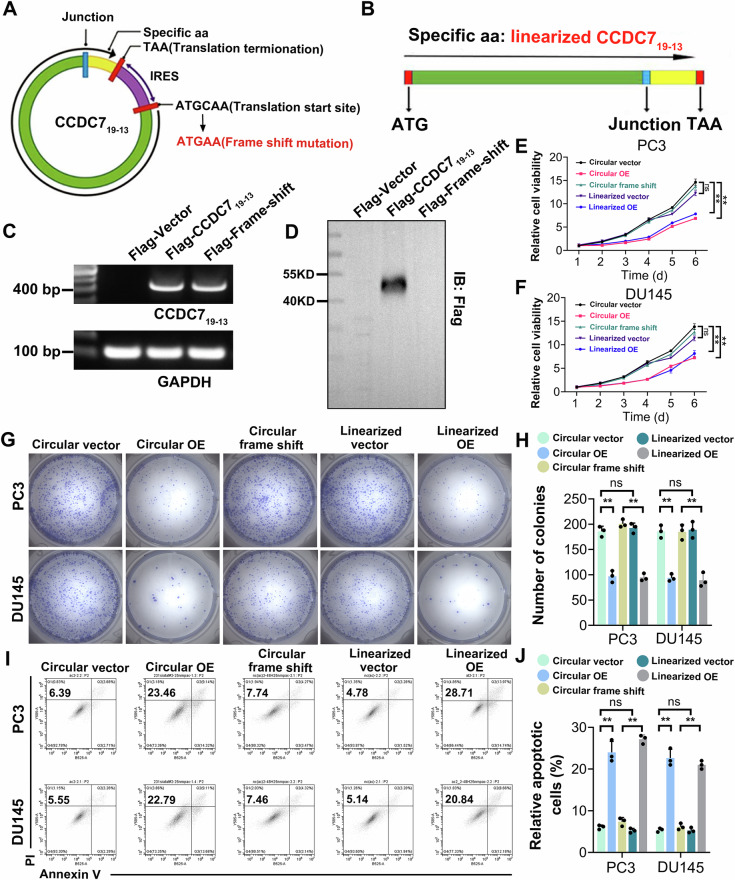
Inhibitory effects of the circular structure and protein-coding potential of CCDC7_19−13_ on prostate cancer. **A** Schematic representation of circular CCDC7_19−13_ expression construct. The circular construct includes a specific amino acid sequence (aa) with a translation start site (ATGCAA) and termination site (TAA), along with an IRES element to facilitate translation. A frame shift mutation (ATGAA) is introduced to study the effects of altered CCDC7 expression. The ability of CCDC7_19−13_ to form a circular structure is essential for its biological function through protein encoding. **B** Diagram showing the linearized form of the CCDC7 construct, highlighting the specific amino acid sequence, start site (ATG), and termination site (TAA). **C** Amplification of CCDC7 (400 bp) and GAPDH (100 bp) in cells transfected with Flag-Vector, Flag-CCDC7_19−13_ and Flag-Frame-shift constructs. GAPDH serves as a loading control. **D** Immunoblotting with anti-Flag antibody confirms the expression of Flag-CCDC7_19−13_ in the corresponding transfected cells. **E**, **F** Cell viability assay in PC3 and DU145 cells. Cells were transfected with Circular vector, Circular OE, Circular frame shift, Linearized vector, and Linearized OE constructs. **G** Representative images of colonies formed by PC3 and DU145 cells transfected with the indicated constructs, stained with crystal violet. **H** The number of colonies formed by PC3 and DU145 cells transfected with different constructs. **I** Flow cytometry analysis of apoptosis. Representative flow cytometry plots showing Annexin V and PI staining in PC3 and DU145 cells transfected with the indicated constructs. **J** Histogram analysis of the percentage of apoptotic cells. Statistical significance is indicated: **p < 0.01, ***p < 0.001, ns not significant.

Current knowledge indicates that chimeric RNAs primarily function through three mechanisms: encoding fusion proteins, miRNA sponging, and protein binding [8, 36]. Chimeric RNAs involved in miRNA sponging and protein binding generally act as non-coding RNAs (ncRNAs) [37]. In contrast, chimeric RNAs that undergo cap-independent translation exert their biological effects mainly through the polypeptides or proteins they encode, leveraging their protein-coding potential to impact cellular functions. Using a pair of divergent primers spanning its splicing site, we confirmed the presence of CCDC7_19-13_ in prostate cancer (PCa) cells and observed that it resisted RNase R digestion, a characteristic feature of circular RNAs (Supplementary Fig. S4A–D). First, open reading frame (ORF) analysis indicated that CCDC7_19-13_ contains essential protein-coding elements, including an internal stop codon (TAA) positioned downstream of the junction site, an internal start codon (ATG), and an internal ribosome entry site (IRES) located between the start and stop codons (Fig. 3A, B, Supplementary Fig. S4E, F). Additionally, IRES activity assays demonstrated that the ID index score for CCDC7_19-13_ was significantly higher than that of other protein-coding circular RNAs, such as circFBXW7 and circZNF609, suggesting enhanced ribosomal binding potential (Supplementary Fig. S4G). Furthermore, while circular RNAs are generally considered more stable, actinomycin D-based half-life analysis revealed that CCDC7_19-13_ exhibited a half-life of ~12 h, similar to protein-coding circular RNAs like GAPDH, circFBXW7, and circZNF609. In contrast, the half-life of the traditional circular RNA circITCH, known to function as a miRNA sponge, exceeded 24 h (Supplementary Fig. S4H).

Given that most ncRNAs primarily reside within the nucleus and regulate transcription, we conducted nucleo-cytoplasmic separation experiments and found that CCDC7_19-13_ was predominantly localized in the cytoplasm, aligning with its protein-coding function rather than transcriptional regulation (Supplementary Fig. S4I, J). Immunofluorescence analysis further confirmed that CCDC7_19-13_ was mainly positioned around the nucleus, resembling Golgi localization (Supplementary Fig. S5A). Additionally, CCDC7_19-13_ overexpression in DU145 cells showed predominant nuclear perinuclear localization (Supplementary Fig. S5B). This suggests that CCDC7_19-13_ is processed within the Golgi and subsequently trafficked to the cell membrane. To investigate whether CCDC7_19-13_ is secreted, we performed an ELISA assay on the cell culture supernatant, which confirmed protein secretion (Supplementary Fig. S5C, D). Western blot analysis following supernatant lyophilization detected a band corresponding to the expected size, further validating the presence of CCDC7_19-13_ peptides (Supplementary Fig. S5E).

To differentiate between its ncRNA-related and protein-coding functions, we introduced a frameshift mutation by deleting a nucleotide upstream of the start codon, which theoretically should not impact ncRNA activity.

As a negative control, we cloned the same ORF plus a 3× FLAG tag into a linear vector (Fig. 3A, B). RT-qPCR and agarose electrophoresis confirmed that both wild-type and frameshift mutant CCDC7_19-13_ were significantly overexpressed (Fig. 3C). However, Western blot analysis revealed an absence of FLAG signal in the frameshift mutant (Fig. 3D). Next, we transfected PC3 and DU145 cells with these constructs to assess their impact on cell viability and apoptosis. Notably, the frameshift mutant did not influence proliferation or apoptosis in prostate cancer cells. In contrast, both wild-type and linearized CCDC7_19-13_ significantly reduced prostate cancer cell viability and enhanced apoptosis (Fig. 3E–J). These findings suggest that the tumor-suppressive ability of CCDC7_19-13_ is primarily attributed to its encoded protein rather than an ncRNA-mediated mechanism.

### CCDC7_19-13_-encoded protein functions through extracellular secretion

**Fig. 4:**
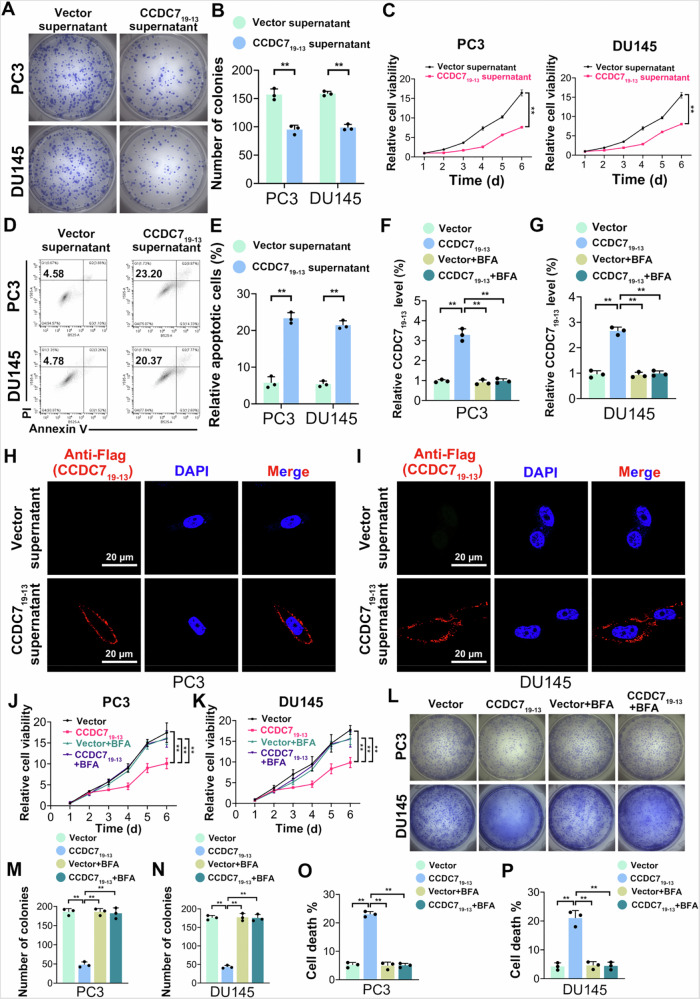
CCDC7_19-13_ encodes a protein that exerts its function extracellularly. **A** Representative images of colony formation assays in PC3 and DU145 cells treated with supernatant collected from PCa cells transfected with Vector or CCDC7_19-13_. Colonies were stained with crystal violet. **B** Quantification of the number of colonies formed by PC3 and DU145 cells. Data represent the mean ± SD from three independent experiments. **C** Relative cell viability of PC3 and DU145 cells treated with supernatant from PCa cells transfected with Vector or CCDC7_19-13_ over a 6-day period, assessed using the CCK-8 assay. Data represent the mean ± SD from three independent experiments. **D** Flow cytometry analysis of apoptosis in PC3 and DU145 cells treated with supernatant from PCa cells transfected with Vector or CCDC7_19-13_. **E** Quantification of apoptotic cells in PC3 and DU145 cells. **F**, **G** ELISA measuring CCDC7_19-13_ levels in PC3 and DU145 cells transfected with Vector, CCDC7_19-13_, or supernatant from cells treated with Brefeldin A (BFA) to block protein secretion. **H**, **I** Immunofluorescence analysis of CCDC7_19-13_ expression in PC3 and DU145 cells treated with supernatant from PCa cells transfected with Vector or CCDC7_19-13_. Cells were stained with anti-Flag antibody (red) and DAPI (blue) to visualize nuclei. **J**, **K** Relative cell viability of PC3 and DU145 cells treated with supernatant from PCa cells transfected with Vector, CCDC7_19-13_, or supernatant from cells treated with BFA, over a 6-day period, assessed using the CCK-8 assay. **L** Representative images of colony formation assays in PC3 and DU145 cells treated with supernatant from PCa cells transfected with Vector, CCDC7_19-13_, or supernatant from cells treated with BFA. Colonies were stained with crystal violet. **M**, **N** Quantification of the number of colonies formed by PC3 and DU145 cells. Data represent the mean ± SD from three independent experiments. Quantification of cell death in PC3 (**O**) and DU145 (**P**) cells treated with conditioned medium derived from prostate cancer cells transfected with Vector or CCDC7_19-13_, with or without Brefeldin A (BFA) pretreatment. Cell death was measured by 7-AAD staining followed by flow cytometry.

Having established that the CCDC7_19-13_ protein can be secreted extracellularly, we collected the culture media from PCa cells transfected with either the CCDC7_19-13_ construct or empty vector. The supernatant was subsequently used to treat PC3 and DU145 cells to investigate the extracellular effects of the CCDC7_19-13_ protein. Notably, treatment with supernatant from CCDC7_19-13_-transfected cells resulted in a significant reduction in colony formation and cell viability in both PC3 and DU145 cells, compared to those treated with supernatant from vector-transfected cells (Fig. 4A–C). Flow cytometry analysis of apoptosis showed an increased percentage of apoptotic cells in PC3 and DU145 cells treated with CCDC7_19-13_ supernatant compared to Vector supernatant (Fig. 4D, E).

To confirm that the observed effects were due to the secreted protein, we treated the cells with supernatant from PCa cells transfected with Vector, CCDC7_19-13_, or supernatant from cells treated with Brefeldin A (BFA) to block protein secretion (Fig. 4F, G). Immunofluorescence confirmed that this protein was mainly enriched on the cell membrane, suggesting that CCDC7_241aa_ may regulate the progression of PCa via potential receptor-ligand interaction (Fig. 4H, I). Further analysis confirmed that the reduction in cell viability and colony formation in PC3 and DU145 cells treated with CCDC7_19-13_ supernatant was significantly diminished in the presence of BFA (Fig. 4J–N). Additionally, quantification of apoptosis revealed a significant increase in cell death in the CCDC7_19-13_ supernatant-treated group, which was reduced when BFA was used to block protein secretion (Fig. 4O, P).

### CCDC7_19-13_ inhibits prostate cancer progression by inducing ferroptosis

Given that CCDC7_19-13_ functions through encoding a novel chimeric protein in cells, we investigated its downstream molecular targets and signaling pathways in prostate cancer cells by performing high-throughput transcriptome sequencing on PC3 and DU145 cells with stable overexpression of CCDC7_19-13_ compared to control cells (Supplementary Fig. S5F, G). KEGG pathway enrichment analysis of the DEGs in PC3 and DU145 cells overexpressing CCDC7_19-13_ indicated that the DEGs were enriched in biological processes such as ferroptosis, ubiquitination, and lipid peroxidation (Supplementary Fig. S5H, I).

**Fig. 5:**
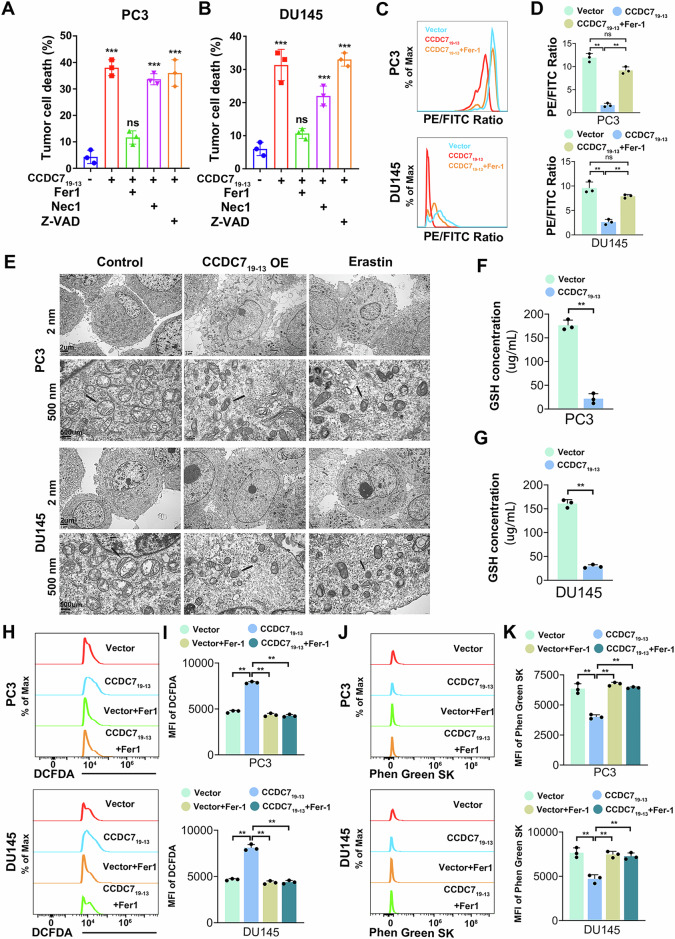
CCDC7_19-13_ inhibits prostate cancer progression by inducing ferroptosis. Quantification of cell death in PC3 (**A**) and DU145 (**B**) cells treated with CCDC7_19-13_, ferroptosis inhibitor (Fer-1), necroptosis inhibitor (Nec-1), or apoptosis inhibitor (Z-VAD). Only Fer-1 significantly rescues cell death, indicating ferroptosis-specific activity. Lipid peroxidation analysis by BODIPY™ 581/591 C11 staining in PC3 (**C**) and DU145 (**D**) cells. A decrease in the PE/FITC fluorescence ratio (red to green shift) indicates increased lipid peroxidation upon CCDC7_19-13_ overexpression, which is reversed by Fer-1 treatment. **E** Transmission electron microscopy of PC3 and DU145 cells treated with Vector, CCDC7_19-13_, or Erastin (10 μM, 24 h). Ferroptotic ultrastructural features such as condensed mitochondria and reduced cristae are visible in CCDC7_19-13_ and Erastin groups. Quantification of intracellular GSH concentrations in PC3 (**F**) and DU145 (**G**) cells. CCDC7_19-13_ significantly reduces GSH levels, consistent with ferroptotic stress. Flow cytometry histograms (**H**) and quantification (**I**) of DCFDA staining show increased ROS levels in PC3 and DU145 cells overexpressing CCDC7_19-13_, reversed by Fer-1. Flow cytometry histograms (**J**) and quantification (**K**) of Phen Green SK staining reveal elevated intracellular Fe^2+^ in CCDC7^19-13^-overexpressing PC3 and DU145 cells, also reversible by Fer-1.

To verify the induction of ferroptosis, we examined cell death mechanisms under CCDC7_19-13_ overexpression. Pharmacological inhibition assays showed that tumor cell death induced by CCDC7_19-13_ was significantly reversed by the ferroptosis inhibitor Ferrostatin-1 (Fer-1), but not by the necroptosis inhibitor Nec-1 or the apoptosis inhibitor Z-VAD, indicating ferroptosis-specific effects (Fig. 5A, B). Lipid peroxidation was assessed by BODIPY™ 581/591 C11 staining. A marked decrease in the PE/FITC fluorescence ratio was observed in both PC3 and DU145 cells upon CCDC7_19-13_ overexpression, consistent with elevated lipid peroxidation; this effect was rescued by Fer-1 (Fig. 5C, D). Transmission electron microscopy further demonstrated classical ferroptotic ultrastructural changes, including shrunken mitochondria and reduced cristae, in cells overexpressing CCDC7_19-13_ or treated with Erastin, but not in control cells (Fig. 5E). Quantification of intracellular glutathione (GSH) revealed significant depletion in CCDC7_19-13_-overexpressing cells compared to vector controls, supporting the disruption of redox homeostasis (Fig. 5F, G).

Flow cytometry analysis of ROS levels using DCFDA showed that CCDC7_19-13_ overexpression led to increased reactive oxygen species production, which was reversed by Fer-1, reinforcing the link between ferroptosis and oxidative stress (Fig. 5H, I). Moreover, analysis with Phen Green SK demonstrated elevated intracellular iron levels in CCDC7_19-13_-overexpressing cells, also attenuated by Fer-1 (Fig. 5J, K). Together, these results confirm that CCDC7_19-13_ triggers ferroptosis in prostate cancer cells through a cascade involving iron accumulation, ROS elevation, lipid peroxidation, and GSH depletion. These ferroptotic features collectively explain its tumor-suppressive effects and highlight its therapeutic potential.

### CCDC7_19-13_ interacts with SLC7A11 and modulates ferroptosis pathway in prostate cancer cells

**Fig. 6:**
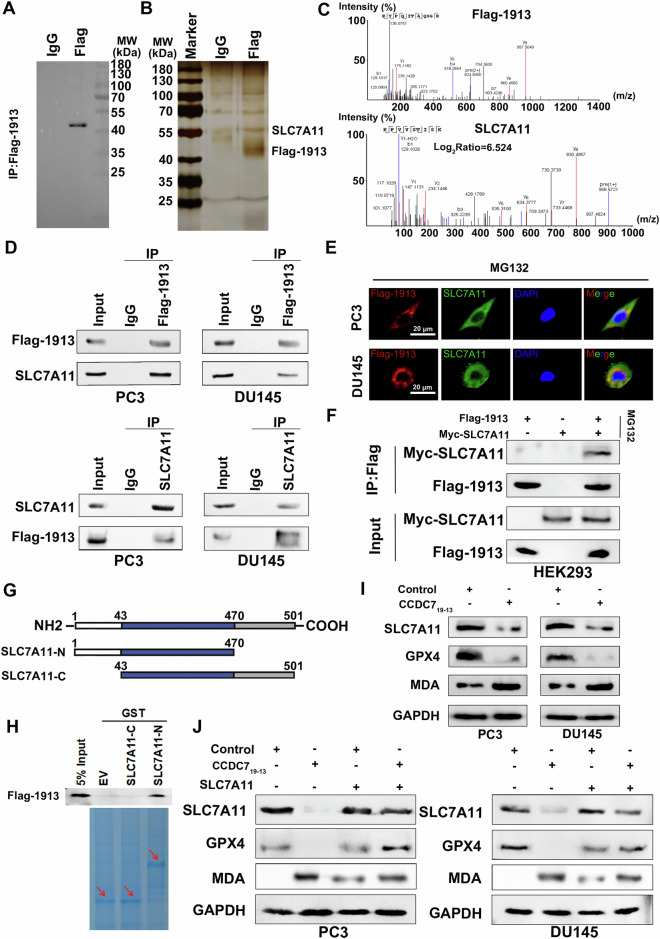
CCDC7_19-13_ interacts with SLC7A11 and modulates ferroptosis pathway in prostate cancer cells. **A** Immunoprecipitation (IP) analysis using anti-Flag antibody demonstrates the presence of Flag-CCDC7_1913_ in PC3 cells, indicating successful overexpression and capture. **B** Co-immunoprecipitation (co-IP) showing the interaction between Flag-CCDC7_19-13_ and SLC7A11. **C** Mass spectrometry analysis confirms the interaction between CCDC7_19-13_ and SLC7A11 with a significant peptide spectrum match. **D** Western blot analysis of IP samples from PC3 and DU145 cells confirming the interaction between CCDC7_19-13_ and SLC7A11. **E** Immunofluorescence microscopy of PC3 and DU145 cells treated with MG132, showing colocalization of CCDC7_19-13_ (red) and SLC7A11 (green), with nuclei stained by DAPI (blue). **F** Co-IP in HEK293 cells co-transfected with CCDC7_19-13_ and Myc-SLC7A11 confirms their interaction. **G** Schematic representation of SLC7A11 truncation mutants (SLC7A11-N and SLC7A11-C) used for mapping the interaction domain with CCDC7_19-13_. **H** GST pull-down assay using GST-tagged SLC7A11 truncation mutants and Flag-CCDC7_19-13_. **I** Western blot analysis of PC3 and DU145 cells showing the effect of CCDC7_19-13_ overexpression on the expression levels of SLC7A11, GPX4, and MDA. **J** Western blot analysis of PC3 and DU145 cells co-transfected with CCDC7_19-13_ and SLC7A11, showing the effect on ferroptosis markers.

To identify downstream targets of CCDC7_19-13_ involved in the induction of ferroptosis, we first utilized functional protein association networks to predict potential interacting proteins (Supplementary Fig. S6A). Mass spectrometry analysis of co-immunoprecipitated proteins identified several interactors of CCDC7_19-13_, with SLC7A11 emerging as a significant candidate (Fig. 6A–C, Supplementary Fig. S6B). Western blot analysis of IP samples from both PC3 and DU145 cells confirmed the interaction between CCDC7_19-13_ and SLC7A11 (Fig. 6D). Immunofluorescence microscopy showed the co-localization of CCDC7_19-13_ and SLC7A11 in PC3 and DU145 cells treated with the proteasome inhibitor MG132, indicating that their interaction occurs in the cytoplasm or cytomembrane (Fig. 6E). Further immunofluorescence analysis revealed that Flag-tagged CCDC7_19-13_ co-localized with SLC7A11 on the cytomembrane of wild-type PC3 and DU145 cells after co-culturing with PC3 and DU145 cells transfected with either the empty vector or Flag-CCDC7_19-13_ plasmid (Supplementary Fig. S6C). Co-IP analysis in HEK293 cells co-transfected with CCDC7_19-13_ and Myc-SLC7A11 further confirmed the interaction between these two proteins (Fig. 6F). To map the interaction domain, we used truncation mutants of SLC7A11 and found that the interaction domain with CCDC7_19-13_ is located in the N-terminal region of SLC7A11, as demonstrated by GST pull-down assays (Fig. 6G, H).

However, Quantitative RT-PCR analysis revealed that overexpression of CCDC7_19-13_ did not significantly alter the mRNA levels of SLC7A11 in PC3 and DU145 cells (Supplementary Fig. S6D, E). Western blot analysis showed that overexpression of CCDC7_19-13_ in PC3 and DU145 cells resulted in decreased expression levels of SLC7A11 and GPX4, along with increased levels of malondialdehyde (MDA), a marker of lipid peroxidation (Fig. 6I). Additionally, co-transfection of PC3 and DU145 cells with CCDC7_19-13_ and SLC7A11 demonstrated that the presence of SLC7A11 modulates the CCDC7_19-13_-induced changes in GPX4 and MDA levels, supporting the role of CCDC7_19-13_ in ferroptosis regulation through its interaction with SLC7A11 (Fig. 6J).

### CCDC7_19-13_ promotes SLC7A11 degradation and modulates ferroptosis in prostate cancer cells

**Fig. 7:**
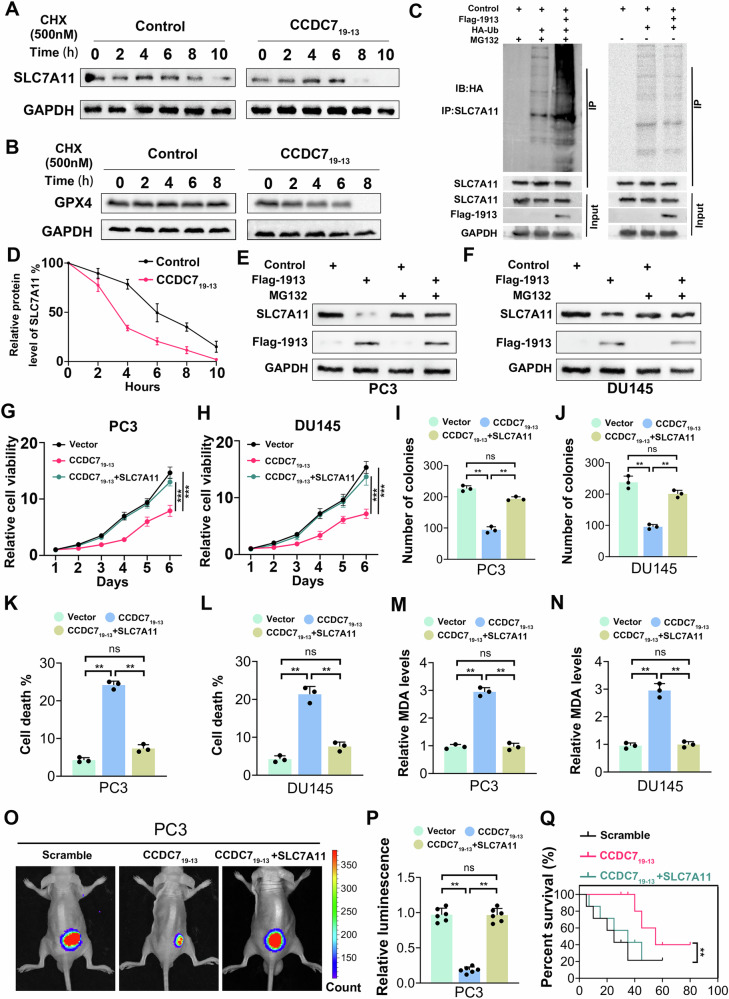
CCDC7_19-13_ promotes SLC7A11 degradation and modulates ferroptosis in prostate cancer cells. **A**, **B** Western blot analysis of SLC7A11 and GPX4 protein levels in PC3 cells treated with cycloheximide (CHX, 500 nM) to block protein synthesis. Cells were transfected with either control vector or CCDC7_19-13_ overexpression. **C** Co-immunoprecipitation (Co-IP) assay showing the ubiquitination of SLC7A11 in HEK293 cells co-transfected with Flag-CCDC7_19-13_ and HA-Ub. Cells were treated with MG132 (10 µM) to inhibit proteasomal degradation. **D** Quantification of SLC7A11 protein levels over time in CHX-treated PC3 cells. Western blot analysis of SLC7A11 protein levels in PC3 (**E**) and DU145 (**F**) cells transfected with control or Flag-CCDC7_19-13_ vector, with or without MG132 treatment. Relative cell viability of PC3 (**G**) and DU145 (**H**) cells transfected as indicated. Colony formation assay of PC3 (**I**) and DU145 (**J**) cells transfected as indicated. Quantification of cell death in PC3 (**K**) and DU145 (**L**) cells as indicated. Cell death was measured by flow cytometry and presented as a percentage. **M**, **N** Relative ferroptosis levels in PC3 and DU145 cells transfected as indicated were determined by MDA levels. **O** Representative bioluminescence images of orthotopic BALB/c mice model injected with PC3 cells transfected as indicated. **P** Quantification of bioluminescence signal from (**O**), representing tumor burden in the mice. **Q** Kaplan–Meier survival curve of mice injected with PC3 cells transfected as indicated.

To investigate the effect of CCDC7_19-13_ on SLC7A11 stability and ferroptosis in prostate cancer cells, we performed a series of experiments using PC3 and DU145 cell lines. We assessed SLC7A11 and GPX4 protein levels in PC3 cells treated with cycloheximide (CHX) to inhibit protein synthesis. Cells transfected with CCDC7_19-13_ showed a more rapid decrease in SLC7A11 and GPX4 levels compared to control cells (Fig. 7A, B). Quantification confirmed accelerated degradation of SLC7A11 in the presence of CCDC7_19-13_ (Fig. 7D). To confirm ubiquitination and proteasomal degradation of SLC7A11, co-immunoprecipitation assays were performed in HEK293 cells co-transfected with Flag-CCDC7_19-13_ and HA-tagged ubiquitin (HA-Ub). Treatment with the proteasome inhibitor MG132 increased ubiquitination of SLC7A11, indicating that CCDC7_19-13_ promotes its degradation via the ubiquitin-proteasome pathway (Fig. 7C). In both PC3 and DU145 cells, CCDC7_19-13_ overexpression significantly reduced SLC7A11 levels, which were partially restored by MG132 treatment, confirming proteasome-mediated degradation (Fig. 7E, F).

In addition, Overexpression of CCDC7_19-13_ significantly decreased cell viability in PC3 and DU145 cells, an effect partially rescued by SLC7A11 co-transfection (Fig. 7G, H). Similarly, colony formation was reduced with CCDC7_19-13_ overexpression but was mitigated by SLC7A11 co-expression (Fig. 7I, J). CCDC7_19-13_ overexpression also significantly increased cell death, as measured by flow cytometry, and this was reduced by SLC7A11 co-expression (Fig. 7K, L). Elevated malondialdehyde (MDA) 4-Hydroxynonenal (4-HNE), and reactive oxygen species (ROS) levels, indicative of increased ferroptosis, were observed with CCDC7_19-13_ overexpression, which were partially rescued by SLC7A11 (Fig. 7M, N, Supplementary Fig. S6F–I). In an orthotopic BALB/c mouse model, bioluminescent imaging showed that CCDC7_19-13_ overexpression significantly reduced tumor growth, an effect partially rescued by SLC7A11 (Fig. 7O, P). Kaplan–Meier survival analysis indicated that CCDC7_19-13_ increased survival, while SLC7A11 co-expression reduced this survival benefit (Fig. 7Q). In summary, CCDC7_19-13_ promotes SLC7A11 degradation via the ubiquitin-proteasome pathway, leading to increased ferroptosis and reduced viability of prostate cancer cells.

### TRIM21 interacts with SLC7A11 and modulates ferroptosis in prostate cancer cells

**Fig. 8:**
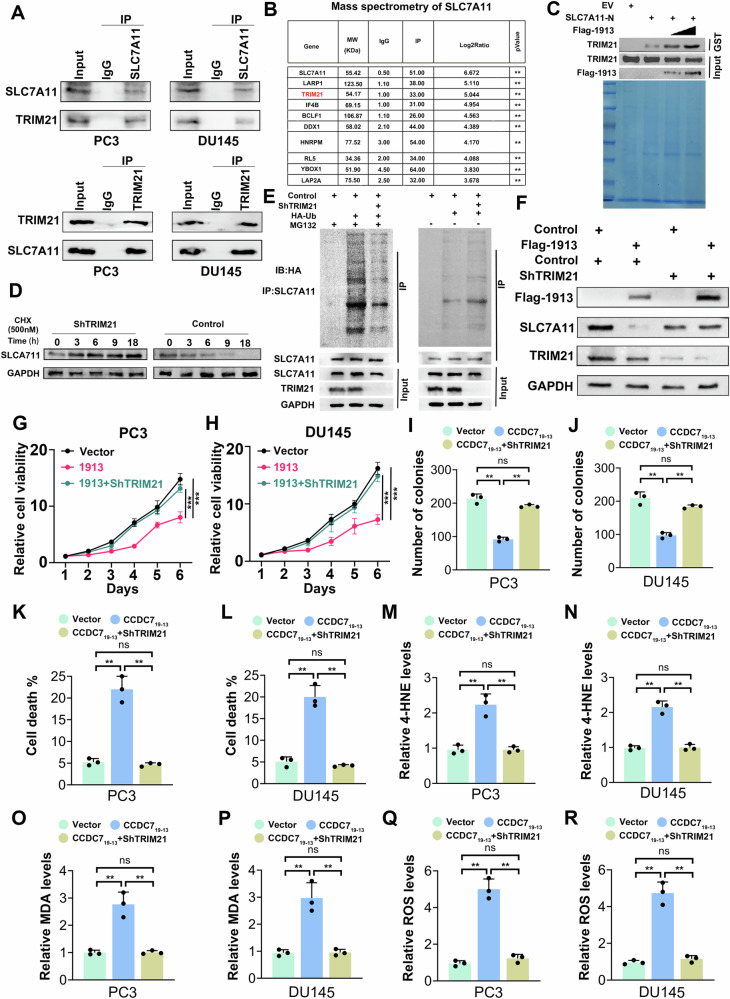
TRIM21 interacts with SLC7A11 and modulates ferroptosis in prostate cancer cells. **A** Co-immunoprecipitation (Co-IP) analysis of TRIM21 and SLC7A11 interaction in PC3 and DU145 cells. SLC7A11 was immunoprecipitated, and TRIM21 was detected by Western blotting. Reverse Co-IP was performed by immunoprecipitating TRIM21 and detecting SLC7A11. **B** Mass spectrometry analysis of SLC7A11 immunoprecipitates from HEK293 cells overexpressing CCDC7_19-13_. The table lists the proteins identified with their corresponding molecular weights (MW) and log2 fold changes (Log2Ratio). **C** GST pull-down assay showing that CCDC7_19-13_ promotes the interaction between TRIM21 and SLC7A11. EV (empty vector) serves as a control. **D** Western blot analysis of SLC7A11 protein levels in PC3 cells treated with cycloheximide (CHX, 500 nM) to block protein synthesis. Cells were transfected with either control or ShTRIM21. Protein levels were assessed at the indicated time points (0, 3, 6, 9, 18 h) post-treatment. GAPDH served as a loading control. **E** Co-IP assay demonstrating the ubiquitination of SLC7A11 in HEK293 cells co-transfected with Flag-CCDC7_19-13_ and HA-Ub, with or without ShTRIM21. Cells were treated with MG132 (10 µM) to inhibit proteasomal degradation. **F** Western blot analysis of SLC7A11 protein levels in PC3 cells transfected as indicated. Relative cell viability of PC3 (**G**) and DU145 (**H**) cells transfected. Colony formation assay of PC3 (**I**) and DU145 (**J**) cells transfected as indicated. Quantification of cell death in PC3 (**K**) and DU145 (**L**) cells transfected with the indicated vectors. Relative ferroptosis levels in PC3 and DU145 cells transfected as indicated were determined by 4-HNE (**M**, **N**), MDA (**O**, **P**), and ROS (**Q**, **R**) levels.

Given that CCDC7_19-13_ is not an E3 ubiquitin ligase, we integrated protein-protein interaction networks (PPI), co-immunoprecipitation (Co-IP), and mass spectrometry data to identify potential E3 ligases mediating SLC7A11 ubiquitination and degradation. Co-IP and Mass spectrometry analysis of SLC7A11 immunoprecipitates from HEK293 cells overexpressing CCDC7_19-13_ identified several interacting proteins. TRIM21 was the most enriched E3 ubiquitin ligase associated with SLC7A11 (Fig. 8A, B; Supplementary Fig. S7A, B). A GST pull-down assay demonstrated that CCDC7_19-13_ enhances the interaction between TRIM21 and SLC7A11 (Fig. 8C). Western blot analysis of PC3 cells treated with cycloheximide (CHX) to block protein synthesis showed that TRIM21 knockdown slowed down the degradation of SLC7A11, indicating that TRIM21 promotes SLC7A11 degradation (Fig. 8D). Co-IP assays in HEK293 cells co-transfected with Flag-CCDC7_19-13_ and HA-Ub, with or without ShTRIM21, showed increased ubiquitination of SLC7A11. MG132 treatment confirmed that TRIM21 mediates SLC7A11 degradation via the proteasome pathway (Fig. 8E).

Western blot analysis in PC3 cells showed that TRIM21 knockdown mitigated the CCDC7_19-13_-induced degradation of SLC7A11 (Fig. 8F). Immunofluorescence analysis demonstrated colocalization of SLC7A11 with the proteasome marker PSMC1/26S in both control and TRIM21-overexpressing cells. TRIM21 overexpression increased the colocalization of SLC7A11 with the proteasome, suggesting enhanced degradation (Supplementary Fig. S7C, D). A Bioluminescence Resonance Energy Transfer (BRET) assay confirmed the interactions between SLC7A11, TRIM21 and CCDC7_19-13_. The normalized relative light units (RLU) indicated significant interactions, particularly with TRIM21, supporting its role in SLC7A11 regulation (Supplementary Fig. S7E).

Moreover, CCDC7_19-13_ overexpression significantly reduced cell viability and colony formation in PC3 and DU145 cells. These effects were partially rescued by TRIM21 knockdown (Fig. 8G–J). Flow cytometry revealed increased cell death with CCDC7_19-13_ overexpression, which was reduced by TRIM21 knockdown (Fig. 8K, L). Elevated levels of 4-HNE and MDA, markers of lipid peroxidation, were observed with CCDC7_19-13_ overexpression, partially mitigated by TRIM21 knockdown (Fig. 8M–P). Similarly, ROS levels increased with CCDC7_19-13_ overexpression and were reduced by TRIM21 knockdown (Fig. 8Q, R). Immunohistochemistry (IHC) analysis of clinical prostate cancer samples showed the expression levels of TRIM21, SLC7A11, and MDA. There was a significant correlation between TRIM21 and MDA levels, and between SLC7A11 and TRIM21 levels (Supplementary Fig. S7F, G). Additionally, the proportion of prostate cancer specimens with high or low SLC7A11 expression stratified by TRIM21 expression levels is shown that a higher percentage of High TRIM21expression specimens had lower SLC7A11 expression (Supplementary Fig. S8A). Kaplan–Meier analysis of progression-free survival further supports these findings. Patients with high TRIM21 or low SLC7A11 expression levels had significantly longer progression-free survival compared to those with low TRIM21 levels (Supplementary Fig. S8B, C). In summary, TRIM21 interacts with SLC7A11 and mediates its degradation, influencing ferroptosis in prostate cancer cells. CCDC7_19-13_ enhances the TRIM21-SLC7A11 interaction, leading to increased SLC7A11 ubiquitination, promoting ferroptosis, and reducing cell viability.

### CCDC7_19-13_/TRIM21 catalyze K48-linked polyubiquitination of SLC7A11 at K12

**Fig. 9:**
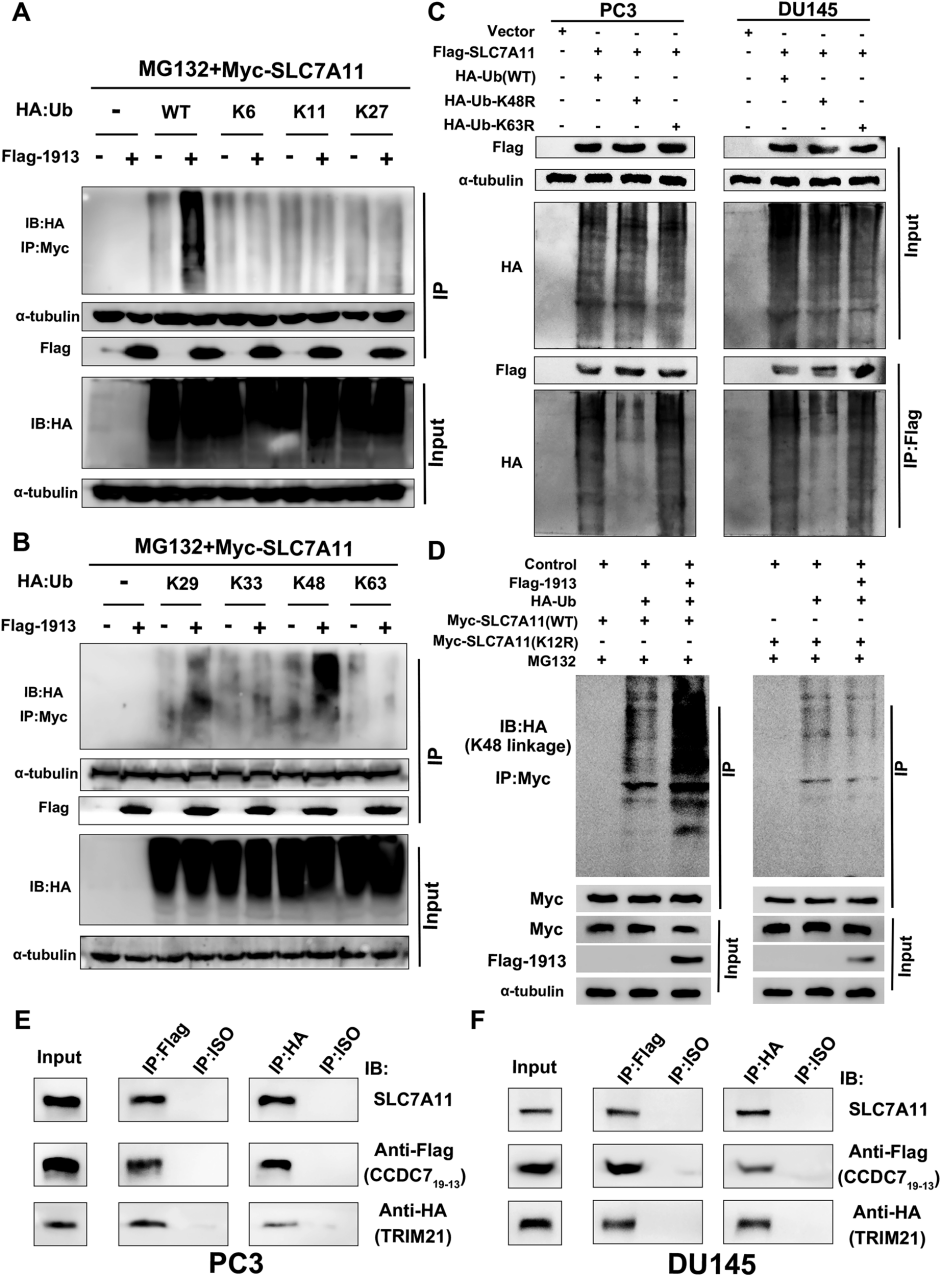
CCDC7_19-13_/TRIM21 catalyze K48-linked polyubiquitination of SLC7A11 at K12. **A** Analysis of ubiquitin linkage types on SLC7A11. PC3 cells were co-transfected with Myc-SLC7A11, Flag-CCDC7_19-13_, and HA-tagged ubiquitin (WT, K6, K11, K27). Cells were treated with MG132 (10 µM) to inhibit proteasomal degradation. Myc-SLC7A11 was immunoprecipitated, and the presence of HA-tagged ubiquitin was detected by Western blotting. **B** Ubiquitin linkage specificity of SLC7A11 in the presence of CCDC7_19-13_. PC3 cells were co-transfected with Myc-SLC7A11, Flag-CCDC7_19-13_, and HA-tagged ubiquitin (K29, K33, K48, K63). **C** Ubiquitination analysis of SLC7A11 in PC3 and DU145 cells transfected with Flag-SLC7A11, HA-tagged ubiquitin (WT, K48R, K63R), and Flag-CCDC7_19-13_. **D** Analysis of SLC7A11 ubiquitination in the presence of wild-type and K12R mutant forms of Myc-SLC7A11. Co-immunoprecipitation (Co-IP) analysis of SLC7A11, CCDC7_19-13_, and TRIM21 in PC3 (**E**) and DU145 (**F**) cells. Immunoprecipitation was performed using anti-Flag, anti-HA, or control IgG antibodies.

Following our initial findings, we further investigated the effect of CCDC7_19-13_/TRIM21 on the ubiquitination of SLC7A11. We first assessed the specific types of ubiquitin linkages on SLC7A11. PC3 cells were co-transfected with Myc-SLC7A11, Flag-CCDC7_19-13_, and various HA-tagged ubiquitin constructs (WT, K6, K11, K27). Cells were treated with MG132 to inhibit proteasomal degradation. Immunoprecipitation of Myc-SLC7A11 followed by Western blotting for HA-tagged ubiquitin revealed that CCDC7_19-13_ promotes K48-linked polyubiquitination of SLC7A11 (Fig. 9A). To determine the specificity of ubiquitin linkages on SLC7A11 in the presence of CCDC7_19-13_, we co-transfected PC3 cells with Myc-SLC7A11, Flag-CCDC7_19-13_, and HA-tagged ubiquitin constructs (K29, K33, K48, K63) and immunoprecipitation followed by Western blotting showed that K48-linked ubiquitination was predominant (Fig. 9B).

Next, we analyzed SLC7A11 ubiquitination in PC3 and DU145 cells transfected with Flag-SLC7A11, HA-tagged ubiquitin (WT, K48R, K63R), and Flag-CCDC7_19-13_. Immunoprecipitation and Western blotting confirmed that CCDC7_19-13_ significantly increased K48-linked polyubiquitination of SLC7A11, which was reduced when K48 was mutated to arginine (Fig. 9C). To identify the specific ubiquitination site on SLC7A11 targeted by CCDC7_19-13_/TRIM21, we used the Site-Plus and GPS-Uber databases to predict potential ubiquitination sites. K12 was identified as a key site. HEK293 cells were co-transfected with Flag-CCDC7_19-13_, HA-Ub, and either wild-type (WT) or K12R mutant SLC7A11. Western blotting showed that K12R mutation significantly reduced K48-linked ubiquitination, confirming K12 as the specific site for CCDC7_19-13_/TRIM21-mediated ubiquitination (Fig. 9D). Co-immunoprecipitation (Co-IP) analysis in PC3 and DU145 cells confirmed the interaction between SLC7A11, CCDC7_19-13_, and TRIM21. Immunoprecipitation was performed using anti-Flag, anti-HA, or control IgG antibodies. Western blotting confirmed the presence of SLC7A11, CCDC7_19-13_, and TRIM21 in the immunoprecipitates, indicating a direct interaction among these proteins (Fig. 9E, F). In summary, our results demonstrate that CCDC7_19-13_ and TRIM21 catalyze K48-linked polyubiquitination of SLC7A11 at K12, promoting its degradation in prostate cancer cells.

### TRIM21 interacts with SLC7A11 and modulates ferroptosis in prostate cancer cells

**Fig. 10:**
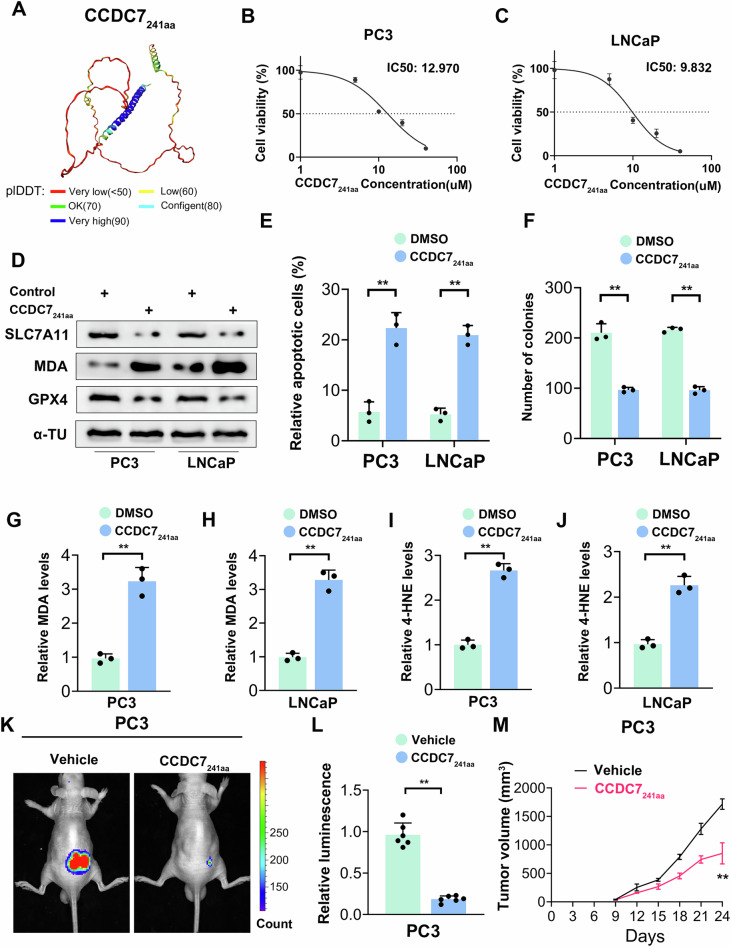
CCDC7_241aa_ induces ferroptosis and reduces tumor growth in prostate cancer cells. **A** Structural model of the CCDC7_241aa_ protein. **B** Dose-response curve illustrating the effect of CCDC7_241aa_ on cell viability in PC3 prostate cancer cells, with an IC50 value of 12.970 µM. **C** Dose-response curve illustrating the effect of CCDC7_241aa_ on cell viability in LNCaP prostate cancer cells, with an IC50 value of 9.832 µM. **D** Western blot analysis of ferroptosis-related proteins (SLC7A11, MDA, GPX4) and α-tubulin (loading control) in PC3 and LNCaP cells treated with CCDC7_241aa_ or control. **E** Flow cytometry analysis of apoptosis in PC3 and LNCaP cells treated with DMSO or CCDC7_241aa_, using Annexin V/PI staining to detect apoptotic cells. **F** Colony formation assay of PC3 and LNCAP cells transfected with DMSO or CCDC7_241aa_. Relative levels of malondialdehyde (MDA) in PC3 (**G**) and LNCAP (**H**) cells treated with DMSO or CCDC7_241aa_. Data are presented as the mean ± SD. **I**, **J** Relative levels of 4-hydroxynonenal (4-HNE) in PC3 (**G**) and LNCAP (**H**) cells treated with DMSO or CCDC7_241aa_. Data are presented as the mean ± SD. **K** In vivo bioluminescence imaging of PC3 tumor-bearing mice treated with vehicle or CCDC7_241aa_. **L** Quantification of bioluminescence signals from (**K**), indicating tumor burden. **M** Tumor volume measurement over time in PC3 tumor-bearing mice treated with vehicle or CCDC_241aa_.

To assess the clinical relevance of targeting CCDC7₂₄₁ₐₐ in prostate cancer therapy, we employed a prokaryotic expression system to generate biologically active recombinant CCDC7₂₄₁ₐₐ protein (Fig. 10A). Initially, we determined the optimal concentration of CCDC7₂₄₁ₐₐ for use in prostate cancer cells via MTT assays. As illustrated in Fig. 10B, C, CCDC7₂₄₁ₐₐ significantly suppressed cell viability in both androgen-insensitive PC3 and androgen-sensitive LNCaP prostate cancer cells in a dose-dependent manner, with calculated IC50 values of 12.970 μM and 9.832 μM, respectively. At the mechanistic level, treatment with CCDC7₂₄₁ₐₐ resulted in a significant reduction in SLC7A11 protein expression in prostate cancer cells (Fig. 10D). Furthermore, CCDC7₂₄₁ₐₐ exposure led to decreased proliferation rates and an increase in apoptosis (Fig. 10E, F, Supplementary Fig. S8D–H). Additionally, ferroptosis-associated markers, including malondialdehyde (MDA) and 4-hydroxynonenal (4-HNE), were markedly elevated (Fig. 10G–J). These findings suggest that CCDC7₂₄₁ₐₐ suppresses prostate cancer cell proliferation and viability in vitro, thereby inducing ferroptosis.

To further investigate the tumor-suppressive properties of CCDC7₂₄₁ₐₐ, we administered either a control vehicle or CCDC7₂₄₁ₐₐ (100 μg/20 μL) directly into tumors in an orthotopic prostate cancer model using BALB/c mice. Bioluminescence imaging revealed a notable reduction in tumor signal intensity in CCDC7₂₄₁ₐₐ-treated mice, indicating a decreased tumor burden. Similarly, tumor volume measurements demonstrated a significant suppression of tumor growth in the CCDC7₂₄₁ₐₐ-treated cohort (Fig. 10K–M). These results collectively demonstrate that CCDC7₂₄₁ₐₐ effectively inhibits tumor growth and activity in prostate cancer, both in vitro and in vivo, by triggering ferroptosis.

To evaluate the systemic safety of CCDC7₂₄₁ₐₐ therapy, we analyzed potential toxicity markers in mice treated with either vehicle or CCDC7₂₄₁ₐₐ. There were no significant alterations in mouse body weight, aspartate aminotransferase (AST) levels, alanine aminotransferase (ALT) levels, total bilirubin (T-BIL), blood urea nitrogen (BUN), or creatinine (CRE) between the groups (Supplementary Fig. S9A–F), indicating no apparent systemic toxicity. Additionally, hematoxylin and eosin (H&E) staining of major organs (liver, kidney, lung, heart, and spleen) revealed no significant histopathological abnormalities (Supplementary Fig. S9G, H). Immunohistochemistry (IHC) analysis of PC3 tumor xenografts further confirmed a substantial increase in MDA expression, a key lipid peroxidation marker associated with ferroptosis, in CCDC7₂₄₁ₐₐ-treated samples. Moreover, Ki67 levels were significantly decreased in the CCDC7₂₄₁ₐₐ-treated group, reinforcing its tumor-inhibitory potential (Supplementary Fig. S9I–L). Overall, these findings indicate that CCDC7₂₄₁ₐₐ exerts a potent tumor-suppressive effect in prostate cancer by inducing ferroptosis, while maintaining a favorable safety profile without causing systemic toxicity.

### CCDC7_241aa_ inhibits tumor growth in PDX models and synergizes with enzalutamide and docetaxel

**Fig. 11:**
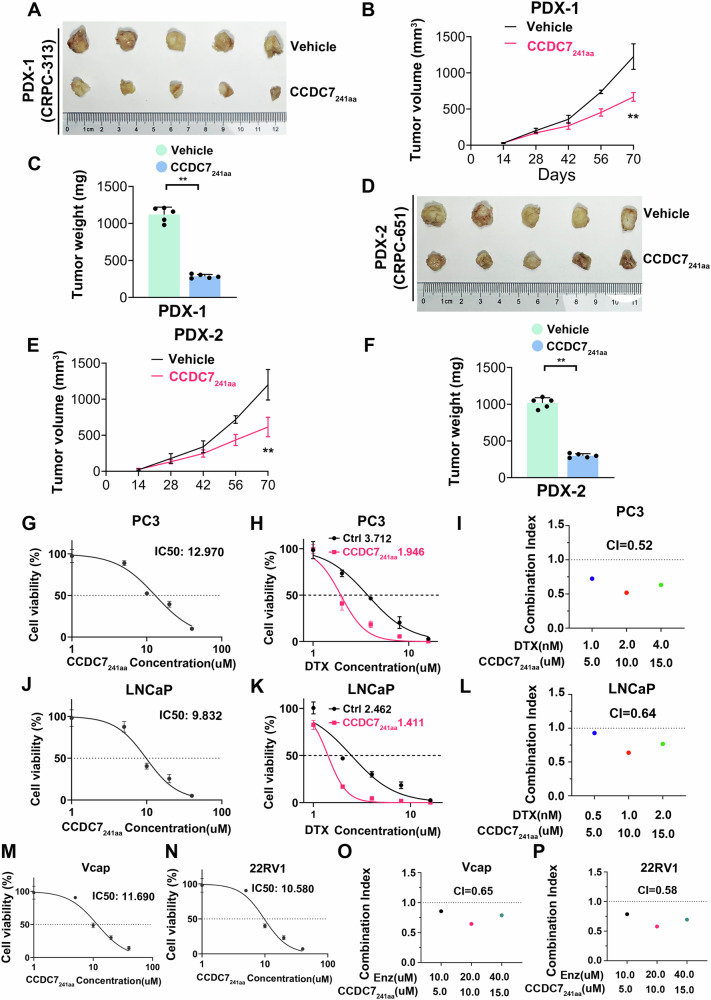
CCDC7_241aa_ inhibits tumor growth in PDX models and synergizes with enzalutamide and docetaxel. **A** Representative images of tumors from PDX-1 (CRPC-313) models treated with vehicle or CCDC7_241aa_. **B**, **C** Tumor volume measurements over time and tumor weight in PDX-1 models treated with vehicle or CCDC7_241aa_. Data are presented as mean ± SD. **D** Representative images of tumors from PDX-2 (CRPC-651) models treated with vehicle or CCDC7_241aa_. **E**, **F** Tumor volume measurements over time and tumor weight in PDX-2 models treated with vehicle or CCDC7_241aa_. **G** Dose-response curve illustrating the effect of CCDC7_241aa_ on cell viability in PC3 prostate cancer cells, with an IC50 value of 12.970 µM. **H** Dose-response curves showing the effect of docetaxel (DTX) on cell viability in PC3 cells pre-treated with control or CCDC7_241aa_. **I** Combination index (CI) plot for PC3 cells treated with a combination of docetaxel (DTX) and CCDC7_241aa_, indicating a synergistic effect (CI < 1). **J** Dose-response curve illustrating the effect of CCDC7_241aa_ on cell viability in LNCaP prostate cancer cells. **K** Dose-response curves showing the effect of docetaxel (DTX) on cell viability in LNCaP cells pre-treated with control or CCDC7_241aa_. **L** Combination index (CI) plot for LNCaP cells treated with a combination of docetaxel (DTX) and CCDC7_241aa_, indicating a synergistic effect (CI < 1). **M** Dose-response curve illustrating the effect of CCDC7_241aa_ on cell viability in VCap prostate cancer cells. **N** Dose-response curve illustrating the effect of CCDC7_241aa_ on cell viability in 22RV1 prostate cancer cells. Combination index (CI) plot for VCap (**O**) and 22RV1 (**P**) cells treated with a combination of enzalutamide (Enz) and CCDC7_241aa_, indicating a synergistic effect (CI < 1). Statistical significance is indicated by asterisks: *p < 0.05, **p < 0.01, and ***p < 0.001.

Recent advancements in surgical techniques and animal experimental models have led to the widespread use of Patient-Derived Xenograft (PDX) models [38, 39]. These models better preserve tumor heterogeneity and stromal cell components, making them valuable for screening targeted drugs for various malignancies. We constructed two PDX models using tumor tissues from CRPC prostate cancer patients (PDX-1:CRPC-313 and PDX-2:CRPC-651). Vehicle or CCDC7_241aa_ was administered intratumorally to these models to further verify the therapeutic effect of CCDC7_241aa_ in advanced prostate cancer. Consistent with the results of CCDC7_241aa_ inhibiting prostate cancer cell viability in vitro, CCDC7_241aa_ significantly inhibited tumor growth compared to the vehicle control in both PDX models (Fig. 11A–F). Additionally, tumors in both PDX models treated with CCDC7_241aa_ showed significantly lower Ki67 expression, a marker for proliferation, and higher MDA expression, a marker for ferroptosis, compared to those treated with the vehicle. These results indicate that CCDC7_241aa_ significantly inhibits tumor growth in CRPC prostate cancer PDX models and provides promising insights for prostate cancer treatment.

Previous research has shown that CCDC7_241aa_ induces ferroptosis in prostate cancer [40]. Several studies have reported that ferroptosis inducers can synergize with existing treatments such as chemotherapy, radiotherapy, immunotherapy, and endocrine therapy to enhance treatment sensitivity [41–44]. To explore whether CCDC7_241aa_ could enhance the sensitivity of first-line treatments for prostate cancer, we performed MTT and IC50 assays to evaluate the effects of different concentrations of first-line prostate cancer treatments (docetaxel and enzalutamide) combined with CCDC7_241aa_ on cell viability. We then calculated the combination index (CI) using CalcuSyn software. We discovered that CCDC7_241aa_ at specific concentrations could synergize with docetaxel in both androgen receptor (AR)-negative prostate cancer cell line PC3 and AR-positive prostate cancer cell line LNCaP (Fig. 11G–L). Additionally, in AR-positive prostate cancer cell lines VCap and 22RV1, CCDC7_241aa_ also synergized with enzalutamide (Fig. 11M–P). These findings suggest that CCDC7_241aa_ inhibits prostate cancer cell viability in vitro by inducing ferroptosis and can synergize with docetaxel and enzalutamide treatments. Combining CCDC7_241aa_ with existing first-line chemotherapy or endocrine therapy may significantly improve therapeutic outcomes for prostate cancer.

## Discussion

Chimeric RNAs, formed by the fusion of exons from two or more originally separate genes through chromosomal rearrangements or post-transcriptional events, have emerged as potential prognostic markers and therapeutic targets in cancer biology. These RNA molecules exhibit unique biological functions, extensively regulating gene expression, participating in signaling networks, and influencing cell fate. Specific fusion genes have been linked to prognosis in various cancers; for example, the EML4-ALK fusion gene in non-small cell lung cancer indicates sensitivity to ALK inhibitors [45], while the TMPRSS2-ERG fusion gene in prostate cancer is associated with poor prognosis [46]. Chimeric RNAs not only serve as biomarkers but also as therapeutic targets, as demonstrated by the success of imatinib targeting the BCR-ABL fusion gene in chronic myeloid leukemia. With the advent of high-throughput sequencing, numerous chimeric RNAs have been identified, and their expression has been correlated with tumorigenesis, progression, prognosis, and treatment resistance in various cancers. In our study, we identified a high-frequency chimeric RNA, CCDC7_19-13_, in the largest sample size Chinese prostate cancer cohort. CCDC7_19-13_ was particularly low in high-grade and castration-resistant prostate cancer (CRPC). Increased expression of CCDC7_19-13_ was independently associated with better prognosis in prostate cancer patients, suggesting its potential role as a prognostic marker and therapeutic target.

Mechanistically, CCDC7_19-13_ encodes a novel fusion protein, CCDC7_241aa_, which acts via autocrine and paracrine signaling to regulate SLC7A11 ubiquitination at K12, mediated by the TRIM21/SLC7A11 axis, thereby inducing ferroptosis in prostate cancer cells. Our study showed that overexpression of CCDC7_19-13_ significantly reduced tumor growth in both in vitro and in vivo models. The recombinant CCDC7_241aa_ protein exhibited robust antitumor activity, inhibiting cell proliferation and promoting apoptosis through the induction of ferroptosis. Notably, the secretion of CCDC7_241aa_ is essential for its paracrine-mediated antitumor activity. Inhibition of ER-to-Golgi trafficking using Brefeldin A (BFA) markedly reduced ferroptosis and cell death induced by CCDC7_241aa_, suggesting that protein secretion is a prerequisite for its bioactivity. This observation aligns with recent findings that vesicle transport and ER stress responses can modulate ferroptotic signaling by affecting lipid peroxidation and iron metabolism [22].

The concept of ferroptosis, an iron-dependent programmed cell death mechanism, has emerged as a promising approach, especially given the heightened iron requirements of cancer cells compared to their normal counterparts [15, 47]. A deeper understanding of ferroptosis mechanisms could lead to innovative therapeutic avenues in cancer treatment. In the context of prostate cancer, our research highlights the role of the protein encoded by the novel chimeric RNA CCDC7_19-13_. This protein interacts with SLC7A11, a crucial player in ferroptosis, and modulates its function through ubiquitination mediated by TRIM21. TRIM21, a RING finger E3 ligase, is known for its versatile roles in cellular processes, but our study uniquely identified it as a central regulator of the ferroptotic pathway [48–50]. We found that TRIM21 targets SLC7A11 for K48-linked ubiquitination at the K12 site, thereby altering iron metabolism and enhancing lipid peroxidation. This process culminates in the induction of ferroptosis, markedly reducing the viability of prostate cancer cells and suggesting a new therapeutic approach. Emerging studies suggest that autophagy, especially ferritinophagy, may facilitate ferroptosis by increasing iron availability. Although we did not observe typical autophagy markers such as LC3-II accumulation in our system, we cannot exclude context-dependent involvement. Further studies using autophagy inhibitors or gene knockdown models (e.g., ATG5/7) are warranted to determine whether CCDC7_19-13_-induced ferroptosis involves autophagic mechanisms [51, 52].

Notably, recombinant CCDC7₂₄₁ₐₐ protein demonstrates synergistic effects when combined with clinically approved therapies such as docetaxel and enzalutamide, two standard treatments for advanced prostate cancer. This synergy, validated through in vitro combination index (CI) analysis, suggests that CCDC7₂₄₁ₐₐ may enhance the efficacy of both chemotherapeutic and hormonal agents, potentially through complementary mechanisms such as ferroptosis induction and modulation of androgen receptor signaling. Such combinatorial regimens may allow for dose reduction of conventional drugs, thereby minimizing associated toxicity and improving therapeutic outcomes. In addition, systemic administration of CCDC7₂₄₁ₐₐ in animal models did not result in observable toxicity, supporting its favorable preclinical safety profile. This highlights the potential of CCDC7₂₄₁ₐₐ not only as a monotherapy but also as a component of rationally designed combination strategies for castration-resistant or chemoresistant prostate cancer.

## Conclusions

Collectively, our research identified the CCDC7_19-13_/TRIM21/SLC7A11 axis as a critical regulator of ferroptosis in prostate cancer, suggesting a novel therapeutic approach. By targeting SLC7A11 for degradation via TRIM21-mediated ubiquitination, this pathway disrupts iron metabolism, leading to selective tumor cell death. This strategy not only enhances our understanding of prostate cancer biology but also suggests the potential for improved treatments by combining ferroptosis induction with other cell death mechanisms. Future work should focus on clinical validation and the exploration of synergistic therapeutic combinations.

## Supplementary information

Original Data Files

Supplementary material

Reproducibility checklis

## Data Availability

All relevant data supporting the conclusions of this study are provided in the main text and/or Supplementary Materials. Datasets generated and/or analyzed during this study can be obtained from the corresponding author upon reasonable request.
