# Supplementary material for: Induction of ferroptosis in prostate cancer by CCDC719-13 via TRIM21-mediated ubiquitination of SLC7A11
Source: Cell Death Differ. 2025 Sep 22;33(3):605–25. doi: 10.1038/s41418-025-01580-x (PMC13035864; doi:10.1038/s41418-025-01580-x)

Figure 3

D

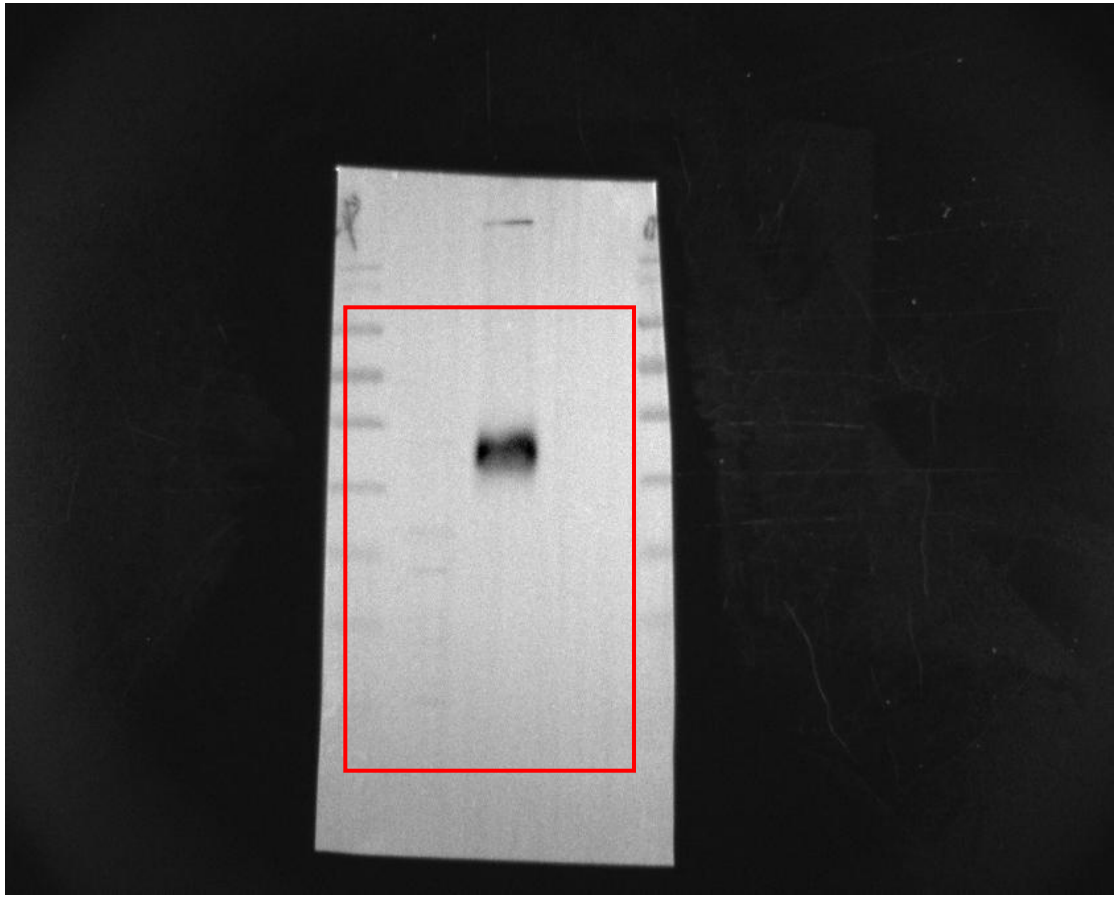

Figure 6

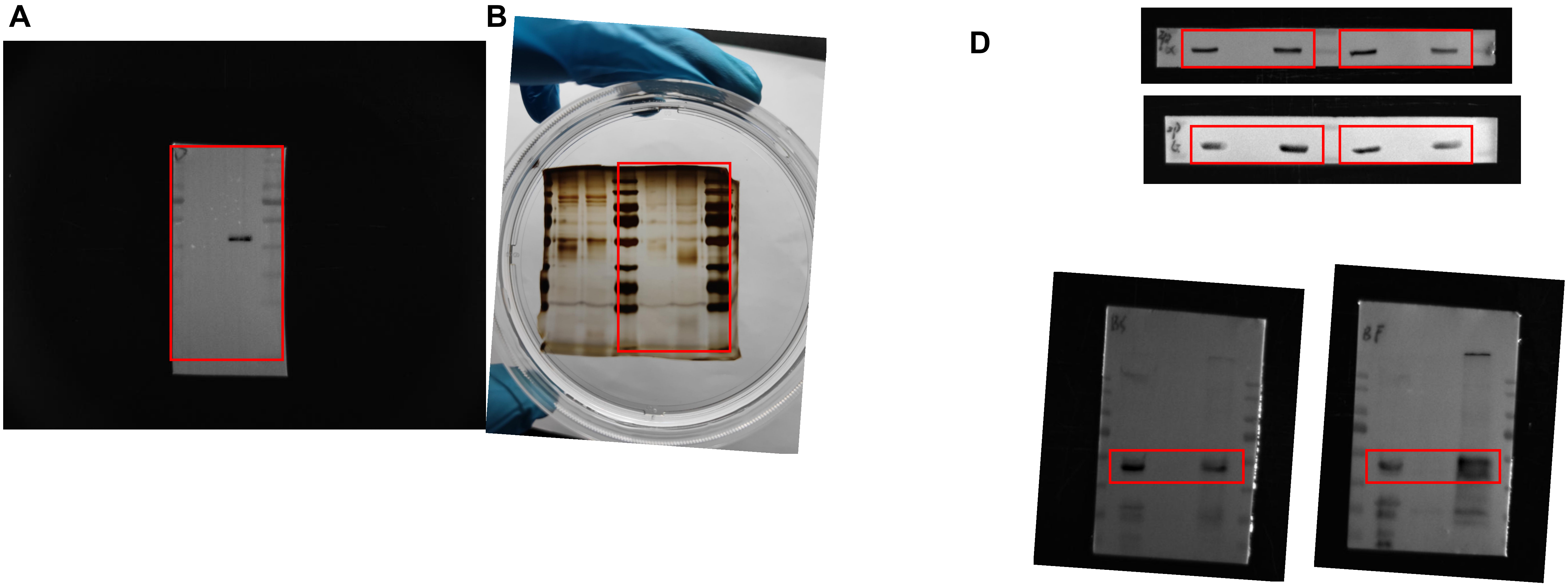

Figure 6

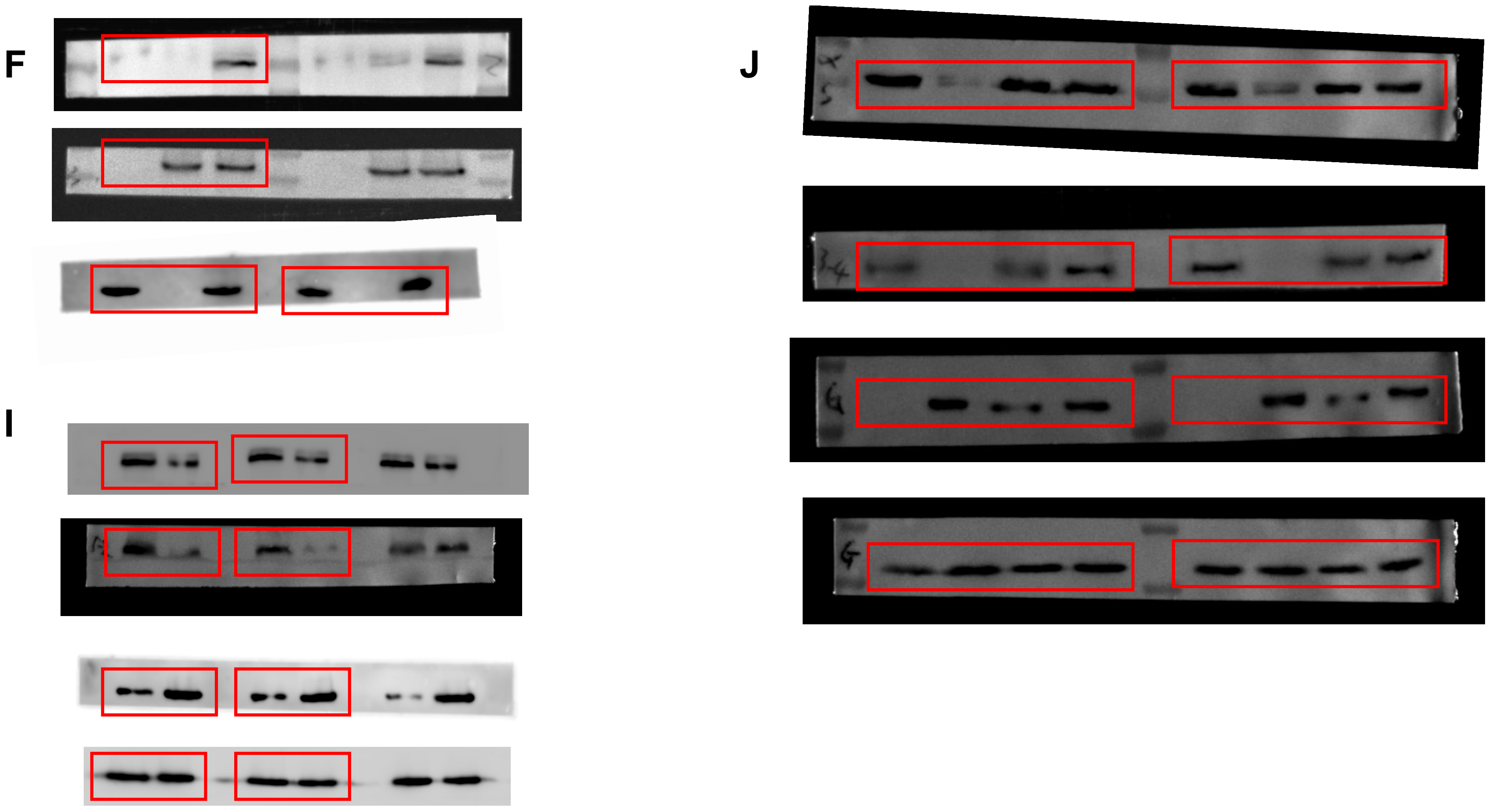

Figure 7

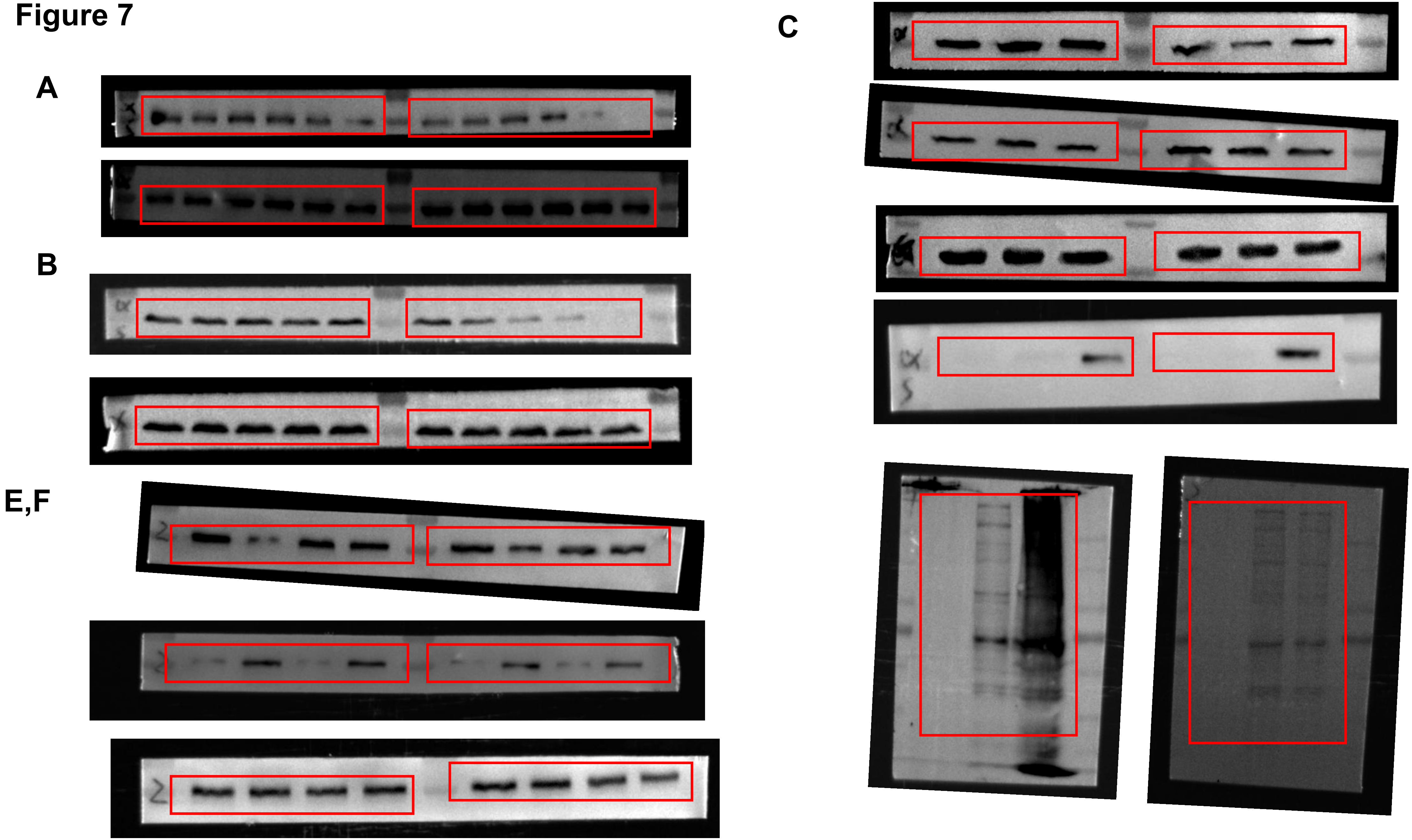

Figure 8

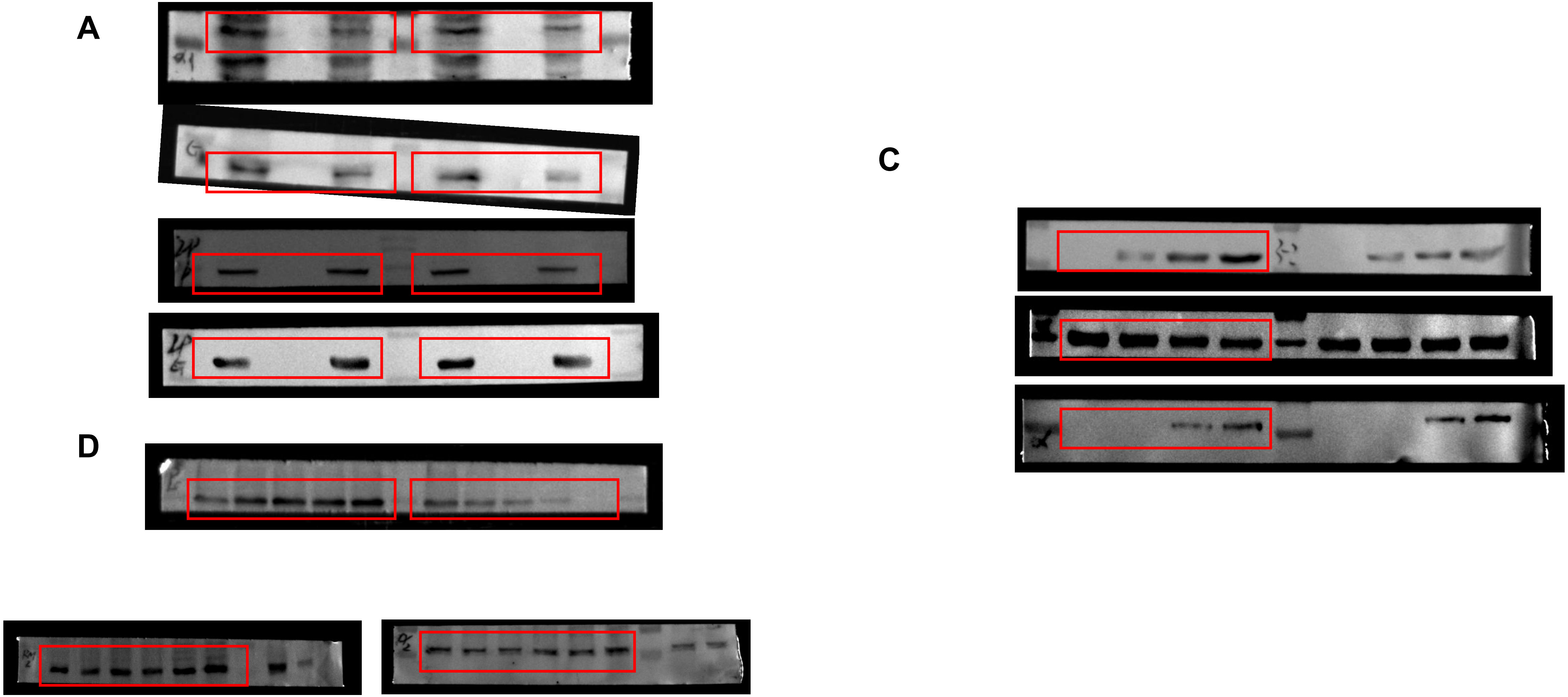

Figure 8

E

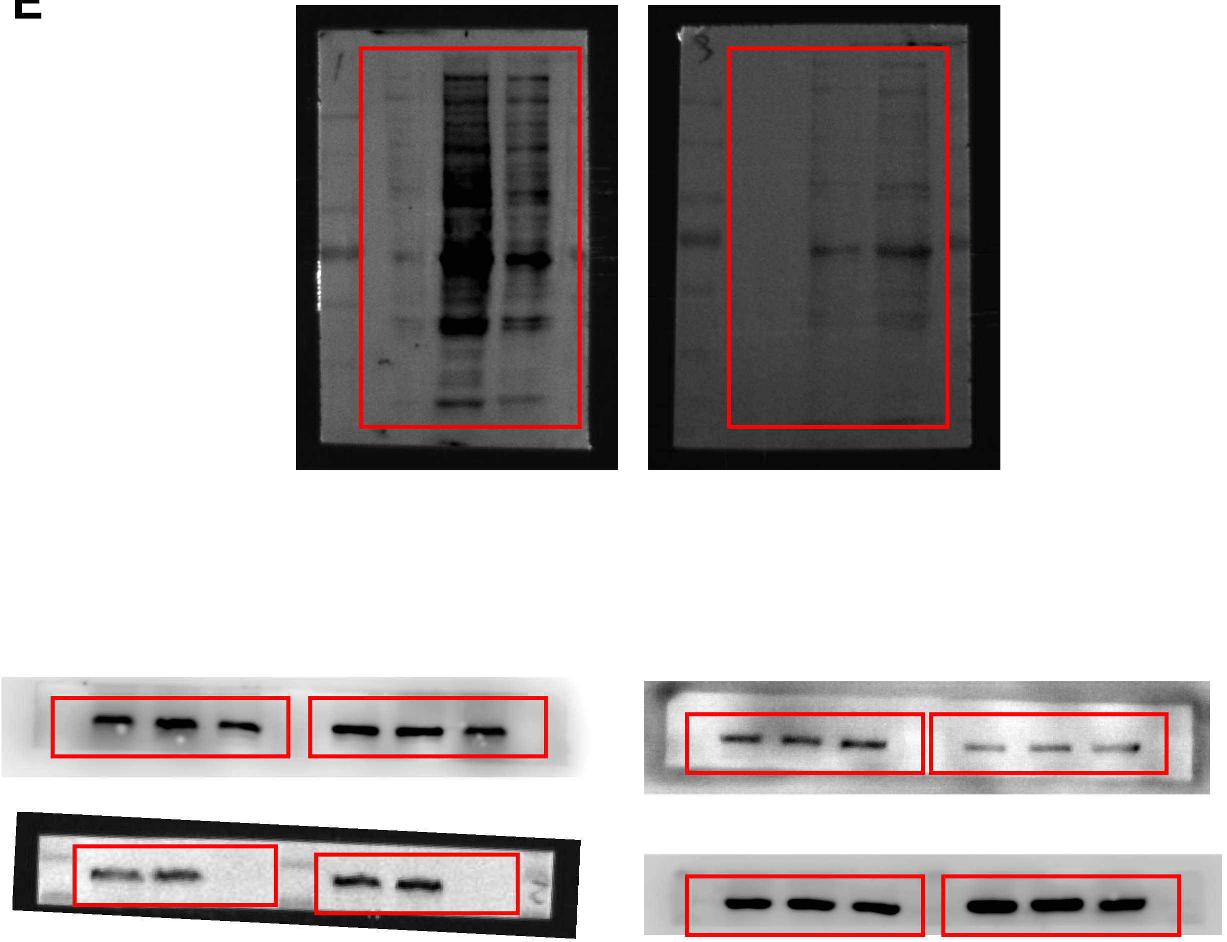

F

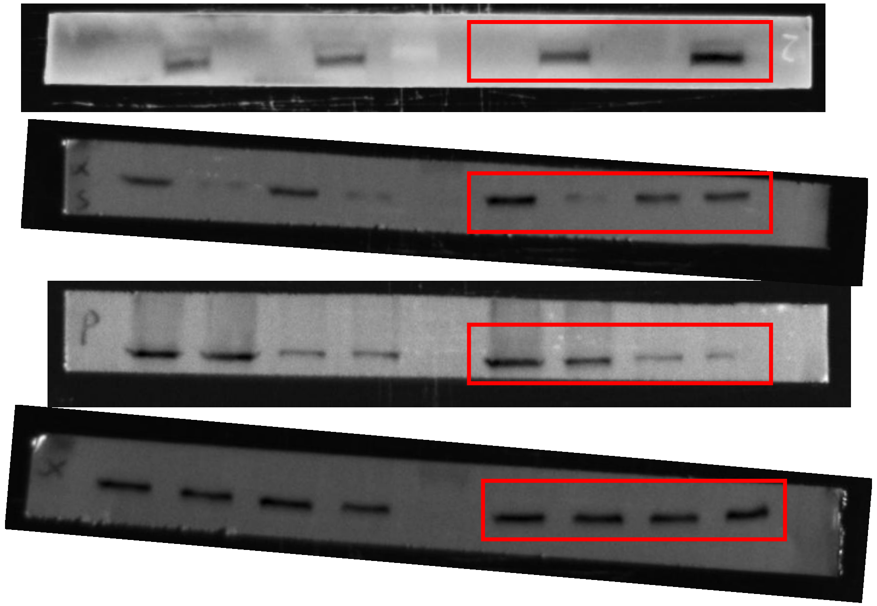

Figure 9

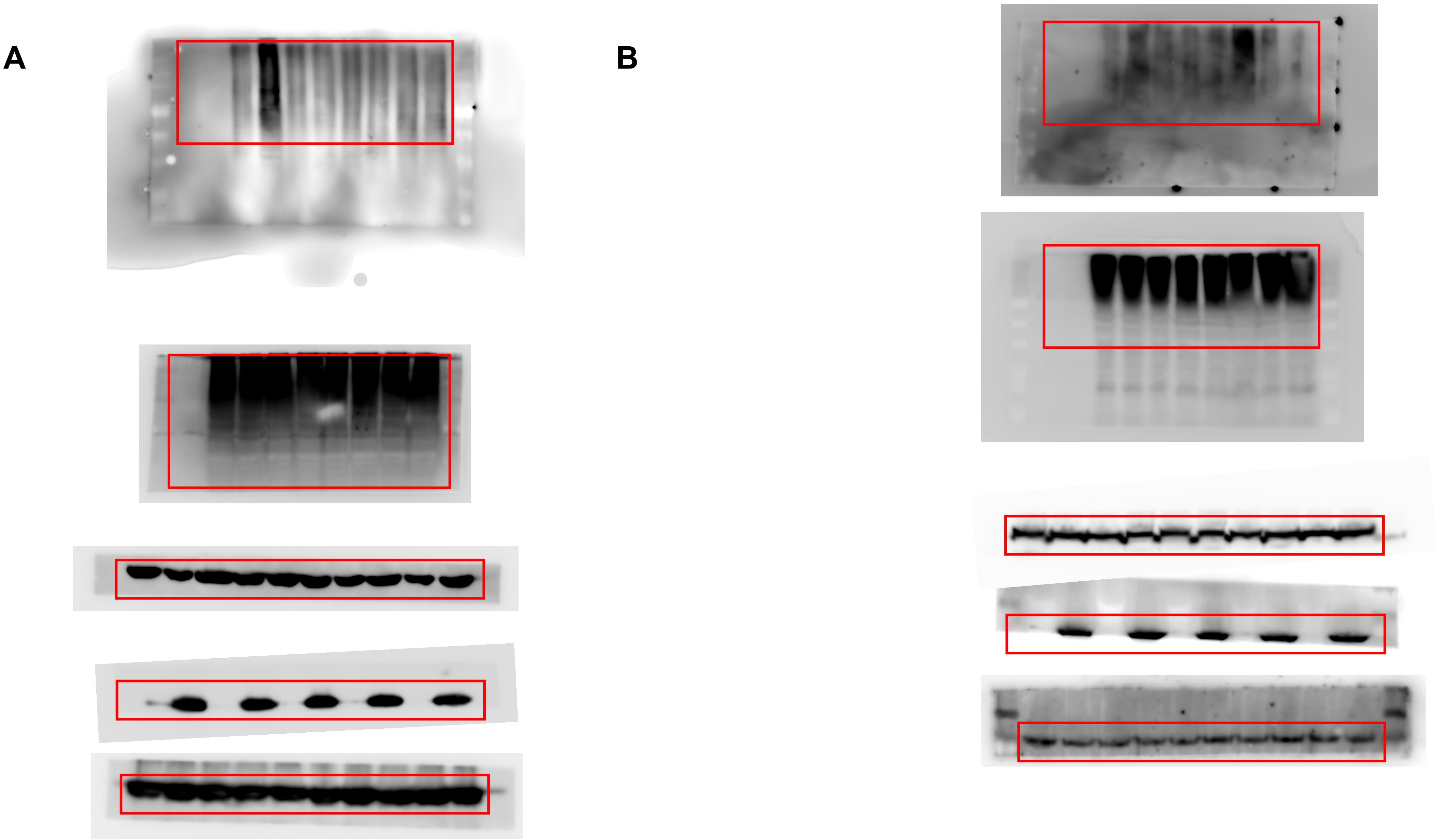

Figure 9

C

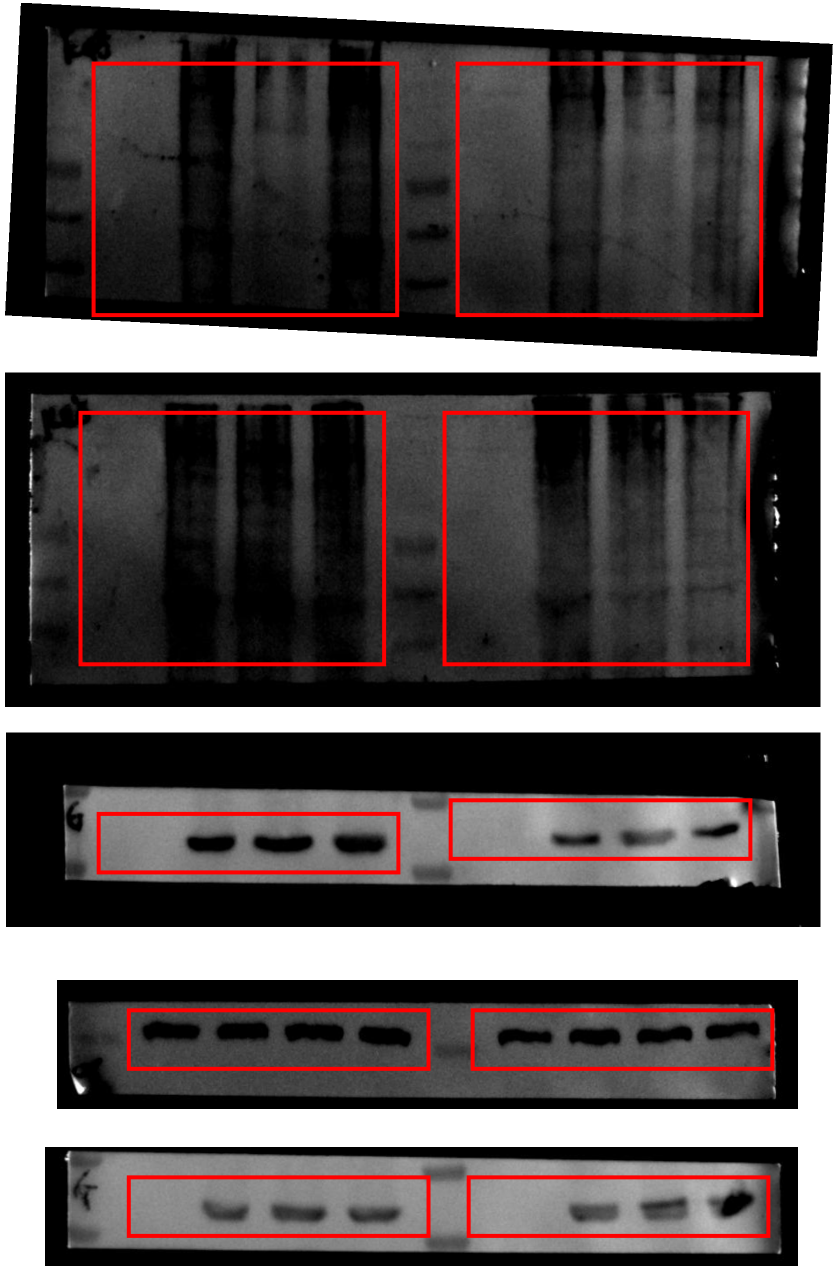

D

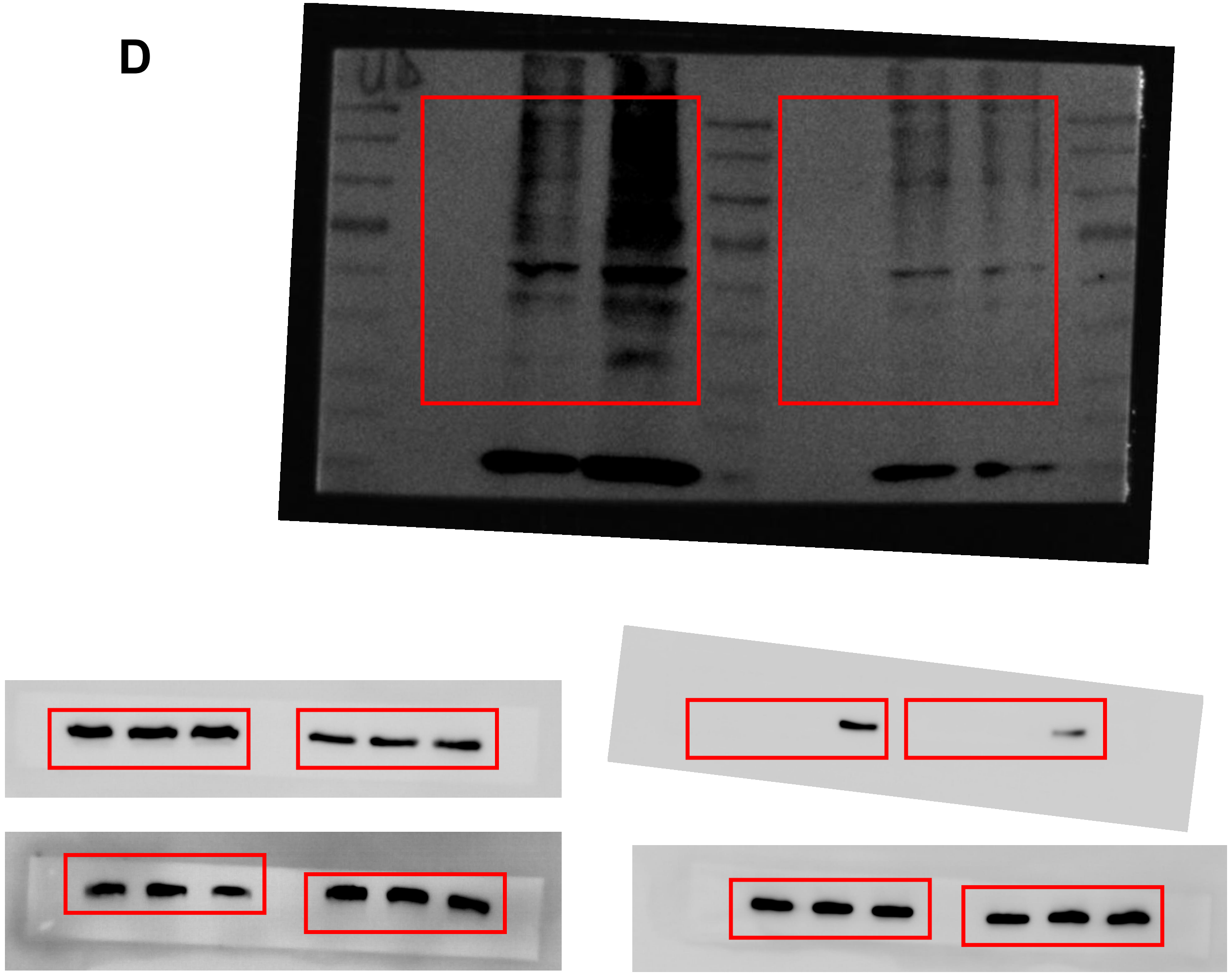

Figure 9

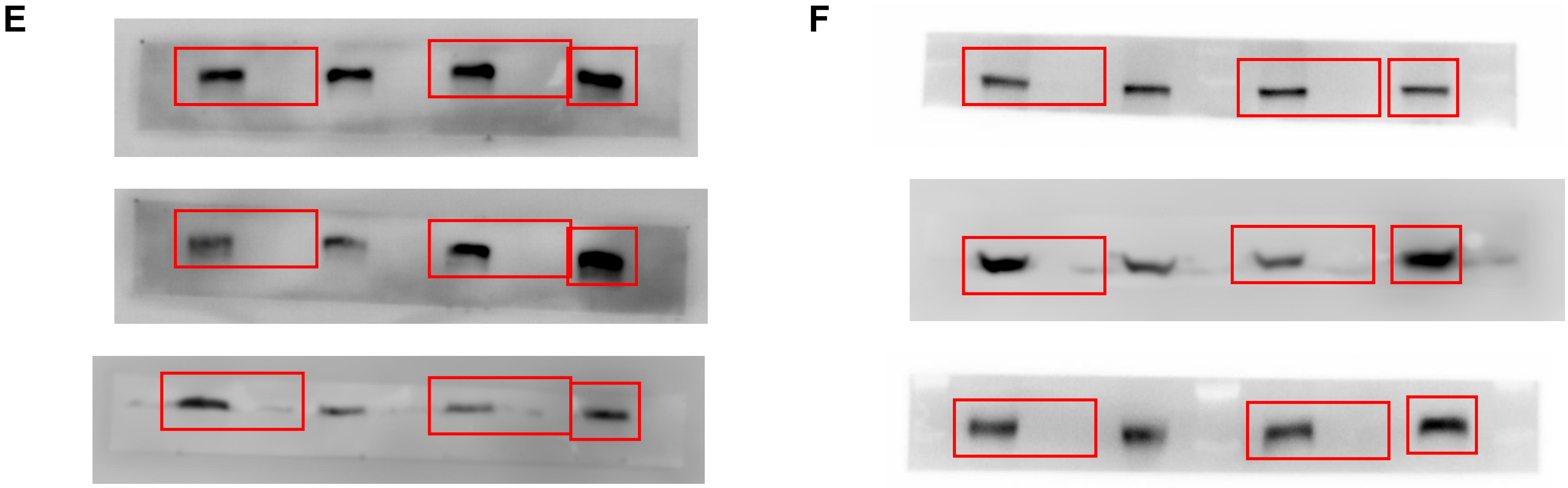

Figure S1

J

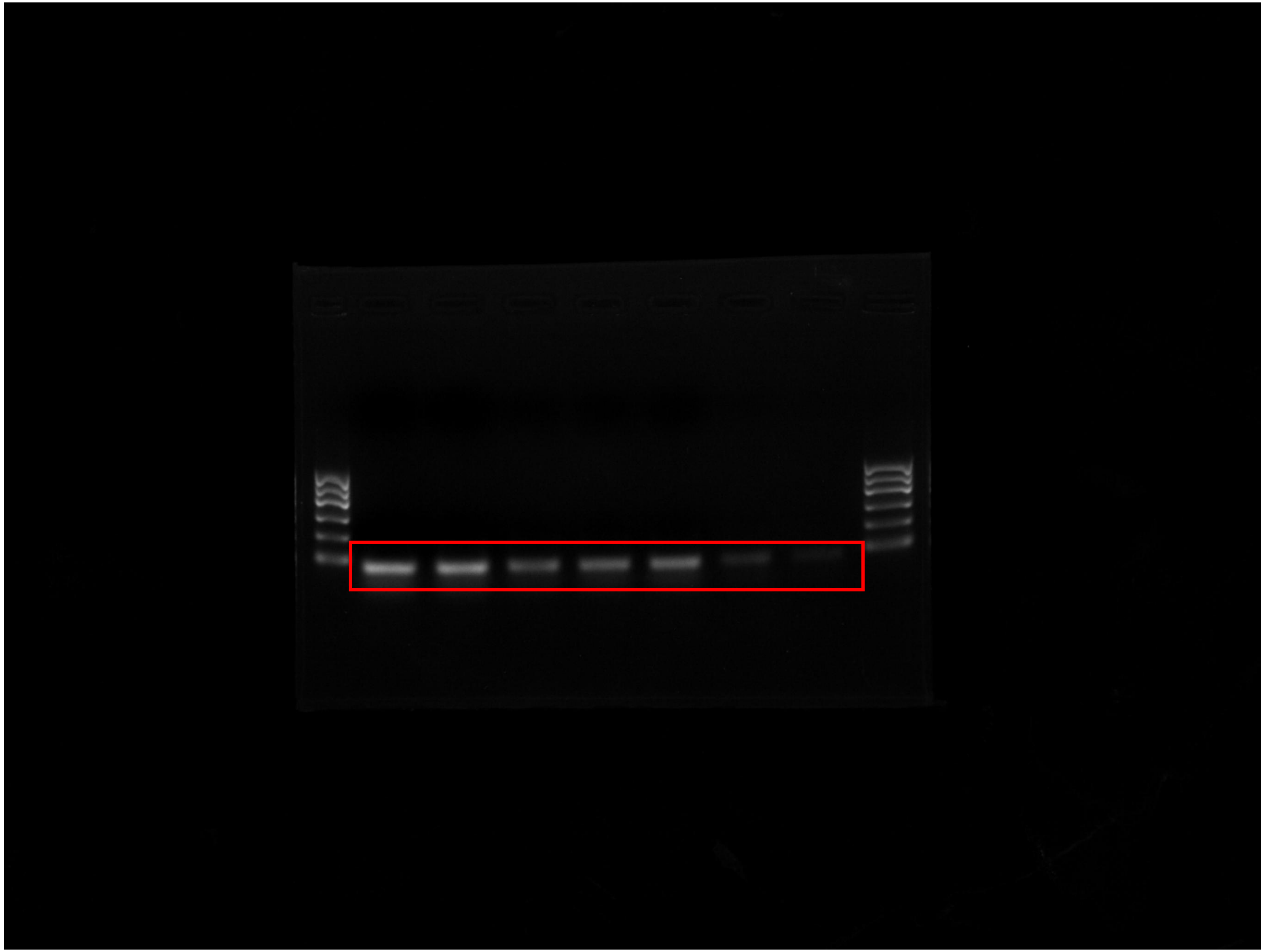

Figure S4

A,B

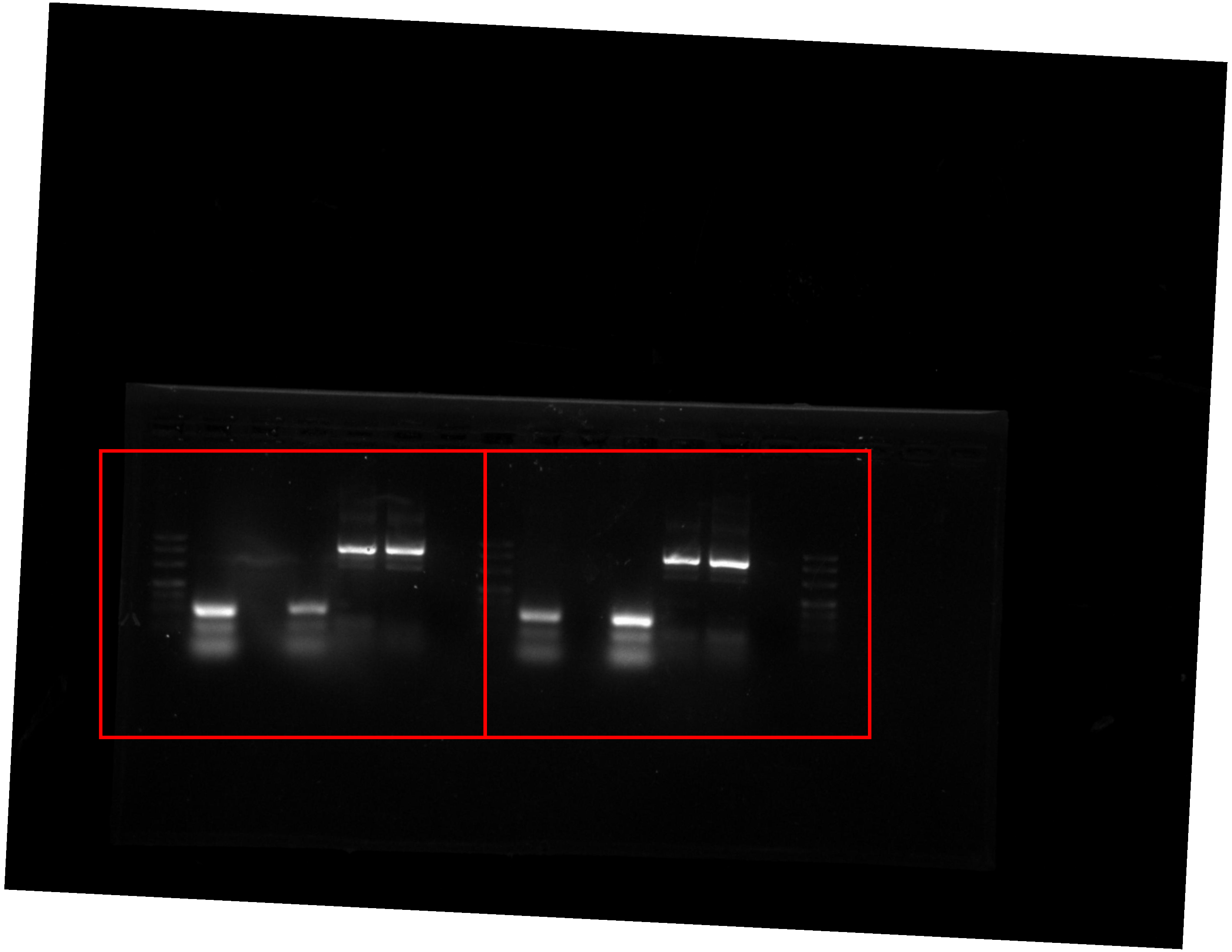

Supplement: Supplementary file 1 — Original Data Files [file 41418_2025_1580_MOESM1_ESM.pdf]
