# Supplementary material for: Induction of ferroptosis in prostate cancer by CCDC719-13 via TRIM21-mediated ubiquitination of SLC7A11
Source: Cell Death Differ. 2025 Sep 22;33(3):605–25. doi: 10.1038/s41418-025-01580-x (PMC13035864; doi:10.1038/s41418-025-01580-x)
Supplement: Supplementary file 2 — Supplementary material [file 41418_2025_1580_MOESM2_ESM.docx]

Table 1 Association between CCDC719-13 expression and clinical features of PCA.

| Patients, n(%) | | | |
| --- | --- | --- | --- |
| Characteristics | High Exp | Low Exp | *p* Value |
| Age,median(IQR) | 69(65-72) | 70(66-74) | 0.7602 |
| PSA at diagnosis (ng/ml),  median(IQR) | 19(10-39) | 44(20-98) | **0.0013** |
| Biopsy Gleason score, n |  |  |  |
| ≤7 | 15(50.0) | 7(23.3) | **0.0321** |
| 8-10 | 15(50.0) | 23(76.7) |  |
| cT-stage,n |  |  |  |
| T1-2 | 20(66.7) | 12(40) | **0.0384** |
| T3-4 | 10(33.3) | 18(60) |  |
| cN-stage,n |  |  |  |
| N0 | 25(31.3) | 21(70) | 0.2221 |
| N1 | 5(68.7) | 9(30) |  |
| cM-stage,n |  |  |  |
| M0 | 23(76.7) | 25(83.3) |  |
| M1a | 4(13.3) | 3(10.0) | 0.8081 |
| M1b | 3(10.0) | 2(6.7) |  |
| ISUP grade |  |  |  |
| 1-2 | 12(40.0) | 6(20.0) |  |
| 3-4 | 7(23.3) | 13(43.3) | **0.0316** |
| 5 | 11(36.7) | 11(36.7) |  |
| ADT = androgen deprivation therapy; IQR = interquartile range;  PSA = prostate-specific antigen;ISUP = International Society of Urological Pathology. | | | |

**Table 2 Regression Analysis of CCDC719-13 Expression and Overall Survival in**

**Prostate Cancer Patients of Cohort 1**

| Variable | Univariate | | | Multivariate | | |
| --- | --- | --- | --- | --- | --- | --- |
|  | HR | 95% CI | *P* | HR2 | 95% CI | *P* |
| Age, years (<65/≥65) | 1.826 | 0.618-4.637 | 0.142 |  |  | NA |
| BMI (<24/≥24) | 1.410 | 0.726-3.268 | 0.369 |  |  | NA |
| Gleason score (<7/≥7) | 3.596 | 1.071-11.959 | **0.038** | 0.362 | 0.029-3.250 | 0.330 |
| Tumor Stage ( T1–T2/T3–T4) | 6.213 | 2.150-17.952 | **0.001** | 2.659 | 0.330-21.437 | 0.342 |
| Metastasis (M0/M1) | 12.721 | 4.306-44.138 | **<0.001** | 13.286 | 4.346-68.634 | **<0.001** |
| CCDC719-13 (low/high) | 0.413 | 0.262-0.924 | **0.001** | 0.422 | 1.510-12.046 | **0.010** |

**Table 3 Regression Analysis of CCDC719-13 Expression and Progression Free**

**Survival in Prostate Cancer Patients of Cohort 1**

| Variable | Univariate | | | Multivariate | | |
| --- | --- | --- | --- | --- | --- | --- |
|  | HR | 95% CI | *P* | HR2 | 95% CI | *P* |
| Age, years (<65/≥65) | 1.895 | 0.692-3.232 | 0.307 |  |  | NA |
| BMI (<24/≥24) | 1.414 | 0.569-2.193 | 0.972 |  |  | NA |
| Gleason score (<7/≥7) | 3.249 | 1.058-8.790 | **0.039** | 1.146 | 0.272-4.771 | 0.758 |
| Tumor Stage ( T1–T2/T3–T4) | 4.308 | 1.480-7.393 | **0.003** | 1.162 | 0.306-3.671 | 0.926 |
| Metastasis (M0/M1) | 8.026 | 2.690-18.499 | **<0.001** | 4.868 | 1.682-14.252 | **0.001** |
| CCDC719-13 (low/high) | 0.365 | 0.245-0.868 | **0.004** | 0.325 | 0.224-0.826 | **0.020** |

**Table 4 Regression Analysis of CCDC719-13 Expression and Overall Survival in**

**Prostate Cancer Patients of Cohort 2**

| Variable | Univariate | | | Multivariate | | |
| --- | --- | --- | --- | --- | --- | --- |
|  | HR | 95% CI | *P* | HR2 | 95% CI | *P* |
| Age, years (<65/≥65) | 1.228 | 0.710-2.556 | 0.396 |  |  | NA |
| BMI (<24/≥24) | 1.026 | 0.426-2.569 | 0.967 |  |  | NA |
| Gleason score (<7/≥7) | 1.699 | 1.277-2.914 | **<0.01** | 1.238 | 0.877-2.914 | 0.068 |
| Tumor Stage ( T1–T2/T3–T4) | 2.768 | 0.978-7.832 | 0.056 |  |  | NA |
| Metastasis (M0/M1) | 3.876 | 2.403-7.608 | **<0.001** | 3.276 | 1.823-6.364 | **0.010** |
| CCDC719-13 (low/high) | 0.462 | 0.236-0.892 | **0.002** | 0.512 | 0.306-0.874 | **0.046** |

**Table 5 Regression Analysis of CCDC719-13 Expression and Progression Free**

**Survival in Prostate Cancer Patients of Cohort 2**

| Variable | Univariate | | | Multivariate | | |
| --- | --- | --- | --- | --- | --- | --- |
|  | HR | 95% CI | *P* | HR | 95% CI | *P* |
| Age, years (<65/≥65) | 1.246 | 0.682-2.242 | 0.680 |  |  | NA |
| BMI (<24/≥24) | 1.263 | 0.492-2.634 | 0.826 |  |  | NA |
| Gleason score (<7/≥7) | 1.612 | 1.221-3.286 | **0.048** | 1.092 | 0.565-2.103 | 0.796 |
| Tumor Stage ( T1–T2/T3–T4) | 2.545 | 1.078-6.022 | **0.036** | 1.413 | 0.540-3.698 | 0.481 |
| Metastasis (M0/M1) | 5.233 | 2.678-8.947 | **<0.001** | 3.804 | 1.898-7.641 | **<0.001** |
| CCDC719-13 (low/high) | 0.398 | 0.204-0.886 | **<0.001** | 0.422 | 0.233-0.912 | **0.020** |

**Table 6 The sequences of small interfering RNAs used in this study.**

| **NO.** | **Target** | **Sequence 5 ’-3 ’** |
| --- | --- | --- |
| **1** | **ShSLC7A11** | AATTTGTCCCTCAGCTGTGCA |
| **2** | **ShGPX4** | TTTAAGGATAACTACCTTGGG |
| **3** | **ShTRIM21** | AATGATACCTATTCTGATAGC |
| **4** | **ShSTAT4** | ATAATAACTTTGTAGTCTCGC |

**Table 7. Antibodies and used in this study.**

| **Application** | **Protein or**  **histone**  **modification** | **Manufacturer** | **Cat**  **number** | **Dilution**  **for usage** |
| --- | --- | --- | --- | --- |
| Western blotting | Flag | CST | #14793 | 1:1000 |
| Western blotting | SLC7A11 | CST | #12691 | 1:1000 |
| Western blotting | GPX4 | CST | #52455 | 1:1000 |
| Western blotting | MDA | Abcam | ab243066 | 1:1000 |
| Western blotting | Ubiquitin | CST | #20326 | 1:1000 |
| Western blotting | HA | CST | #3724 | 1:1000 |
| Western blotting | Myc-Tag | CST | #2278 | 1:1000 |
| Western blotting | α-tubulin | Proteintech | 11224-1-AP | 1:5000 |
| Western blotting | GAPDH | Proteintech | 10494-1-AP | 1:3000 |
| Immunohistochemistry | MDA | Abcam | ab243066 | 1:200 |
| Immunohistochemistry | Ki-67 | Proteintech | 27309-1-AP | 1:200 |
| Immunohistochemistry | SLC7A11 | Proteintech | #12691 | 1:200 |
| Immunohistochemistry | Luciferase | Abbkine | 6B8 | 1:200 |
| Co - immunoprecipitation | SLC7A11 | CST | #12691 | 1:50 |
| Co - immunoprecipitation | TRIM21 | CST | #92043 | 1:50 |
| Co - immunoprecipitation | Flag | CST | #14793 | 1:50 |
| Co - immunoprecipitation | IgG | CST | #3900 | 1:500 |
| Elisa | Flag | Abcam | ab285234 |  |

Proteintech (Wuhan, Hubei, China). CST (Danvers, Massachusetts, USA).Abcam

(Cambridge, UK)

**Table 8 The sequences of primers used in this study.**

| **NO.** | **Target** | **Forward (5'-3')** | **Reverse (5'-3')** |
| --- | --- | --- | --- |
| **1** | **CCDC719-13** | ACAAACTTACCAAGCAGCAG | CACAGCTTTCTTTGTTCGAT |
| **2** | **SLC7A11** | TCTCCAAAGGAGGTTACCTGC | AGACTCCCCTCAGTAAAGTGAC |
| **3** | **GPX4** | GAGGCAAGACCGAAGTAAACTAC | CCGAACTGGTTACACGGGAA |
| **4** | **TRIM21** | GTCCTGGAAAGGAGTGAGTCC | CTGAAAGTATCAGCCACGGATT |
| **5** | **U6** | CTCGCTTCGGCAGCACA | AACGCTTCACGAATTTGCGT3 |
| **6** | **β-Tubulin** | TTGGGAGGTCATCAGCGATGAG | AGGCTCCAGATCCACCAGGATG |
| **7** | **β-Actin** | CACCATTGGCAATGAGCGGTTC | AGGTCTTTGCGGATGTCCACGT |
| **8** | **GAPDH** | CAAGGCTGAGAACGGGAAG | TGAAGACGCCAGTGGACTC |


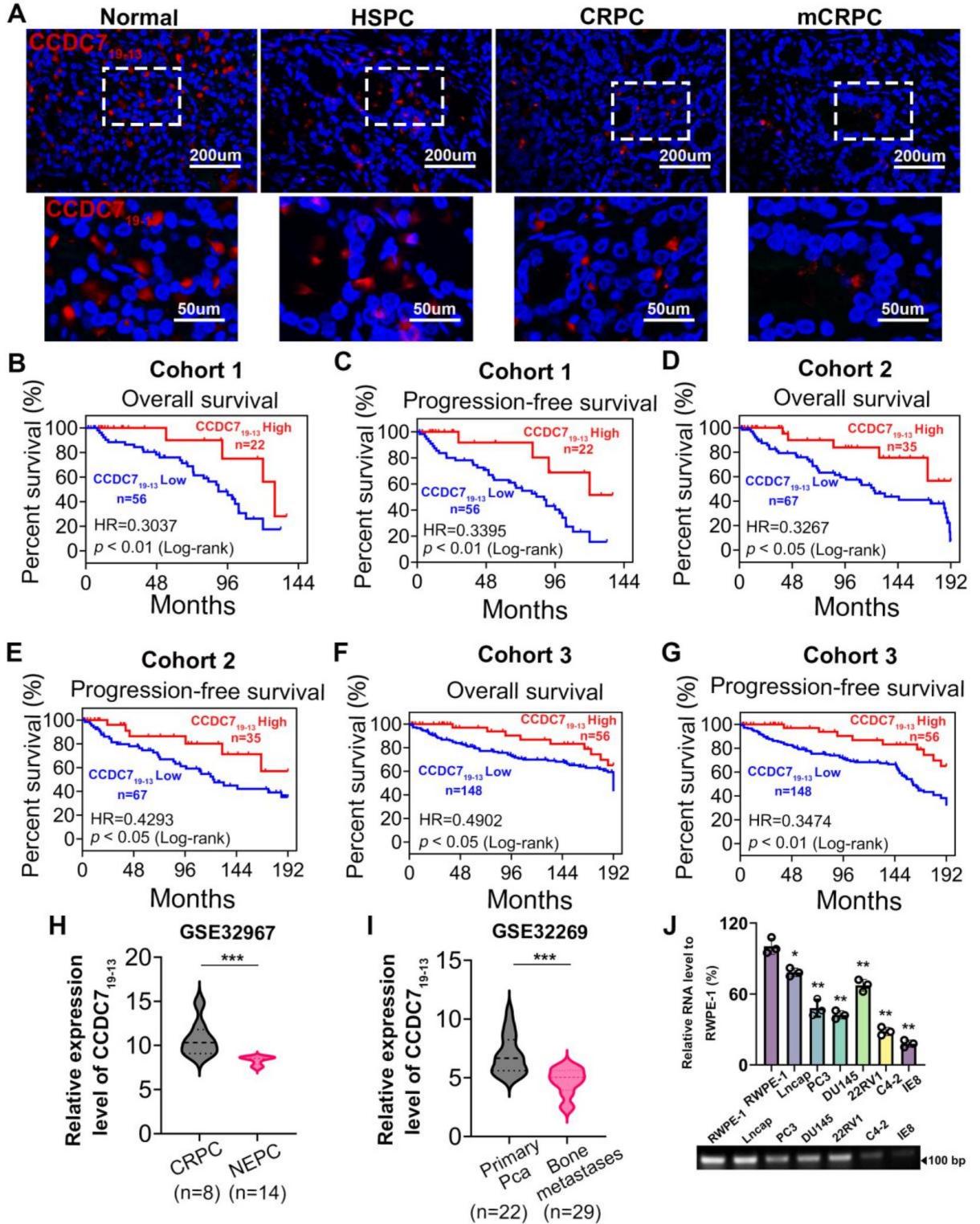
1 **Supplementary figure legends**

2 Supplementary Figure S1. CCDC719-13 was associated negatively with poor

3 prognosis in prostate cancer (PCa).

4

5 (A) Fluorescence In Situ Hybridization (FISH) analysis of CCDC719-13 expression in

6 normal prostate tissue, hormone-sensitive prostate cancer (HSPC), castration-resistant

7 prostate cancer (CRPC), and metastatic CRPC (mCRPC). CCDC719- 13 is shown in red,

8 with nuclear counterstaining in blue. Scale bars: upper panels, 200 μm; lower panels,

9 50 μ m.(B, C) Kaplan-Meier survival curves for (B) overall survival and (C)

10 progression-free survival in Cohort 1.(D, E) Kaplan-Meier survival curves for (D)

11 overall survival and (E) progression-free survival in Cohort 2. (F, G) Kaplan-Meier

12 survival curves for (F) overall survival and (G) progression-free survival in Cohort 3.

13 (H) Violin plot comparing relative expression levels of CCDC719-13 in

14 castration-resistant prostate cancer (CRPC, n=8) and neuroendocrine prostate cancer

15 (NEPC, n=14) from the GSE32967 dataset.(I) Violin plot comparing relative

16 expression levels of CCDC719-13 in primary prostate cancer (n=22) and bone

17 metastases (n=29) from the GSE32269 dataset.(J) Relative RNA levels of CCDC719- 13

18 across different prostate cancer cell lines (PC3, DU145, 22Rv1, C4-2, and LNCaP)

19 compared to normal prostate epithelial cells (RWPE-1). **P* < 0.05 and ***P* < 0.01.

Supplementary Figure S2. Effect of CCDC719-13 on Cell Cycle Progression in Prostate Cancer Cells.


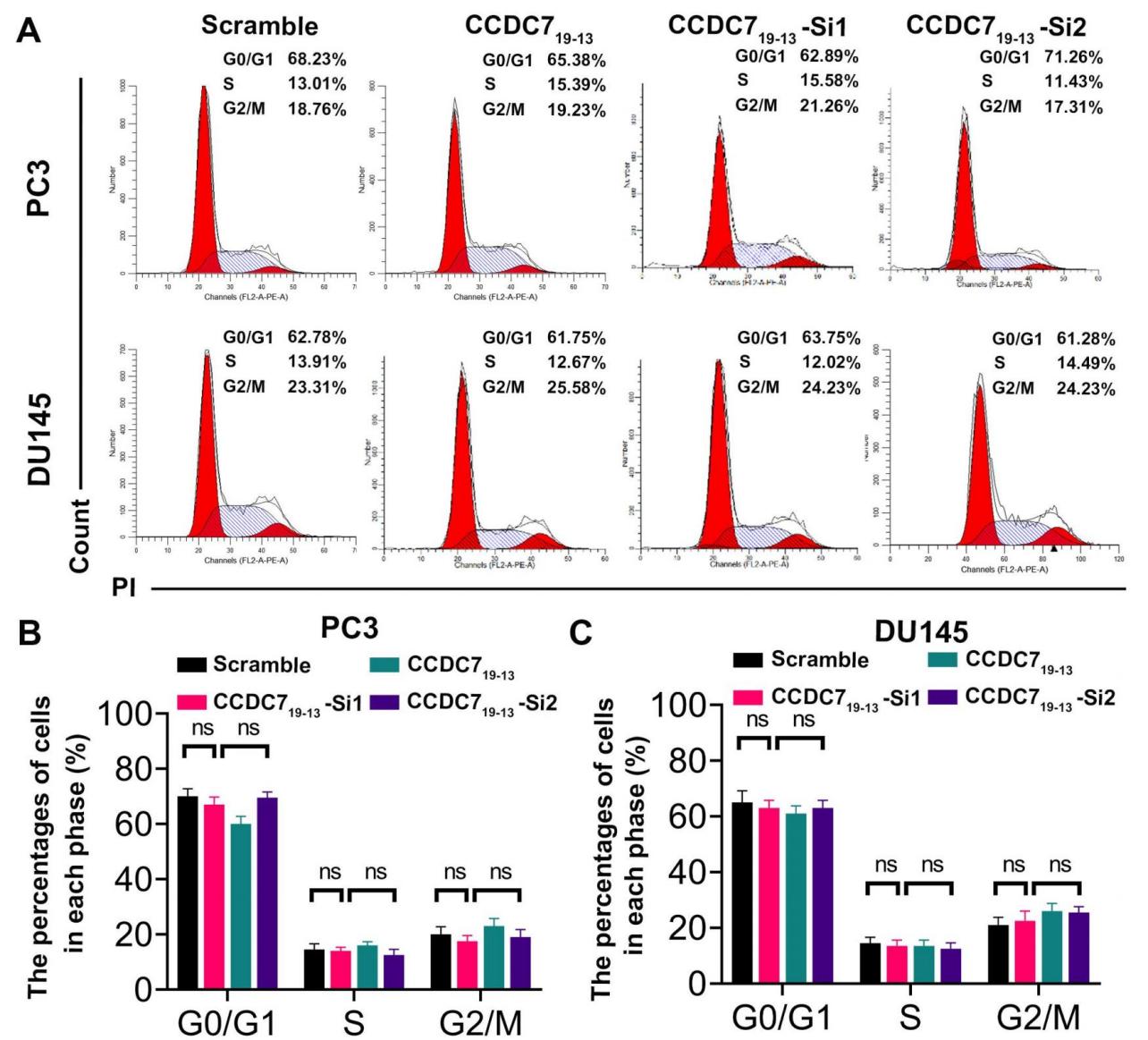


(A) Flow cytometry analysis of cell cycle phases in PC3 and DU145 prostate cancer cells. Cells were transfected with scramble control, CCDC719- 13 overexpression vector, or CCDC719-13-specific siRNAs (CCDC719-13-Si1 and CCDC719-13-Si2). Cell cycle distribution in G0/G1, S, and G2/M phases was determined using propidium iodide (PI) staining. Representative histograms show the percentage of cells in each phase.(B) Quantification of cell cycle phases in PC3 cells. Bar graph representing the percentage of cells in G0/G1, S, and G2/M phases for scramble control, CCDC719-13 overexpression, and CCDC719- 13 knockdown (CCDC719- 13-Si1 and CCDC719- 13-Si2) groups. No significant differences (ns) were observed among the groups.(C) Quantification of cell cycle phases in DU145 cells. Bar graph representing the percentage of cells in G0/G1, S, and G2/M phases for scramble control, CCDC719-13 overexpression, and CCDC719- 13 knockdown (CCDC719- 13-Si1 and CCDC719- 13-Si2) groups. No significant differences (ns) were observed among the groups.

Supplementary Figure S3. Effects of CCDC719-13 on Tumor Growth and Survival in Prostate Cancer Xenografts.


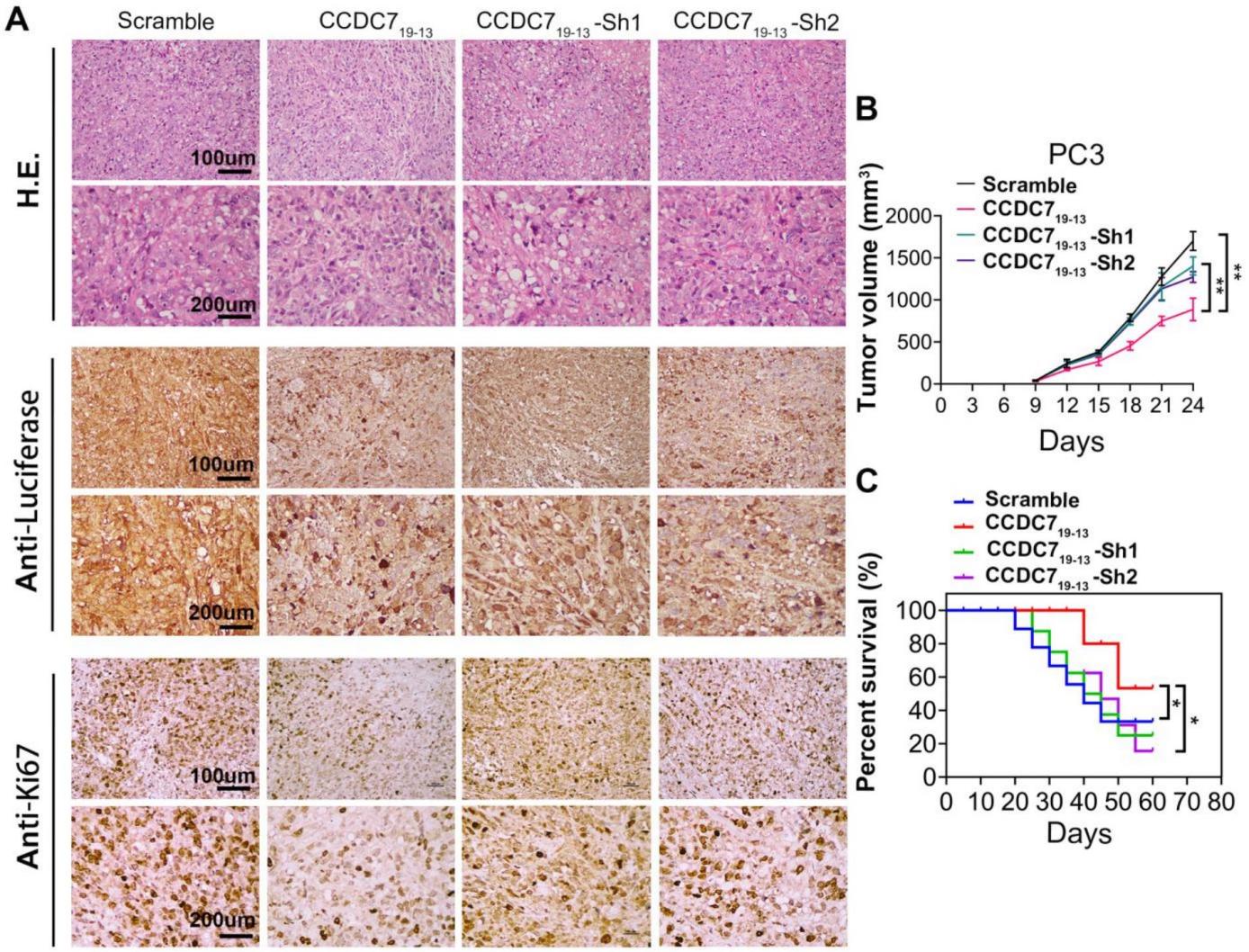


(A) Histological analysis of PC3 tumor xenografts. Hematoxylin and eosin (H.E.) staining, anti-luciferase immunohistochemistry (IHC), and anti-Ki67 IHC of tumor sections from mice injected with PC3 cells transfected with scramble control, CCDC719- 13 overexpression vector, or CCDC719- 13-specific shRNAs (CCDC7_19-13_-Sh1 and CCDC719-13-Sh2). Representative images are shown for each group. Scale bars: 100 μ m (upper panels) and 200 μm (lower panels).(B) Tumor growth curves of PC3 xenografts in mice. Tumor volume was measured over time in mice injected with PC3 cells transfected with scramble control, CCDC719-13 overexpression vector, or CCDC719- 13-specific shRNAs.Data are presented as mean tumor volume ± standard deviation. Statistical significance was determined by two-way ANOVA. **p < 0.01.(C) Kaplan-Meier survival analysis of mice bearing PC3 xenografts for each group.

Supplementary Figure S4. Characterization and Localization of Chimeric RNA CCDC719- 13 in Prostate Cancer Cells


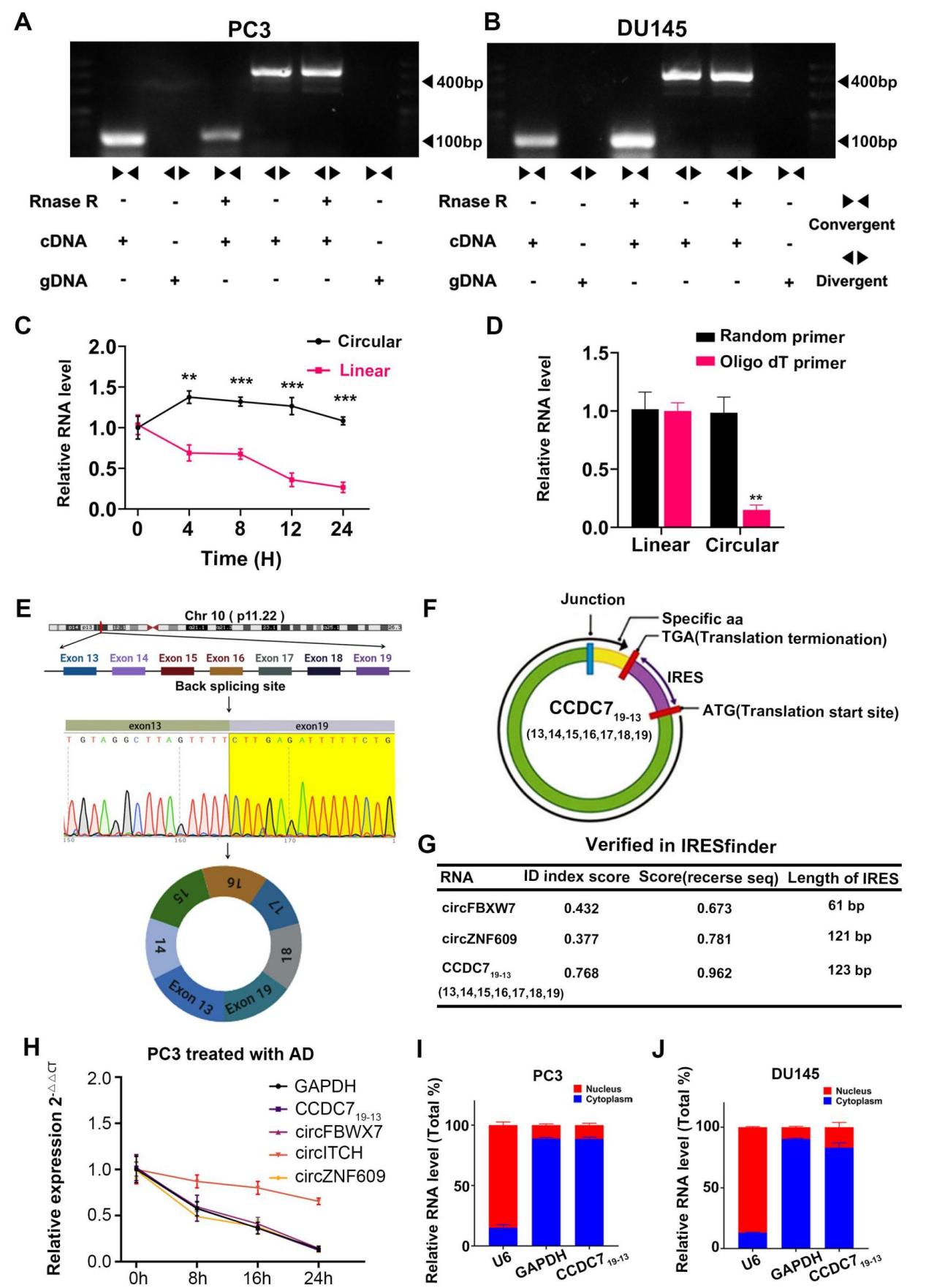


(A, B) RNase R treatment and reverse transcription-PCR (RT-PCR) validation of CCDC719-13 in (A) PC3 and (B) DU145 cells. Agarose gel electrophoresis shows the resistance of circular RNA to RNase R digestion, confirming its circular nature.(C) Stability analysis of CCDC719-13 compared to its linear counterpart over 24 hours post-actinomycin D treatment in PC3 cells. Data are presented as relative RNA levels

(mean ± SD). (D) Relative RNA levels of linear and circular CCDC719- 13 in PC3 cells using random primers and oligo(dT) primers, demonstrating the selective amplification of circular RNA.(E) Schematic representation and sequencing validation of the back-splicing junction of CCDC719-13. The exons involved in forming the circular RNA are shown, with the back-splicing site confirmed by Sanger sequencing.(F) Schematic diagram of the CCDC719-13 structure, including the internal ribosome entry site (IRES), translation start site (ATG), and translation termination site (TGA).(G) IRESfinder analysis of CCDC719-13 and other circular RNAs (circFBXW7, circZNF609) showing the ID index score, score (reverse sequence), and length of the IRES.(H) Relative expression levels of GAPDH, CCDC719-13, circFBXW7, circITCH, and circZNF609 in PC3 cells treated with actinomycin D (AD) over a time course (0h, 8h, 16h, 24h). Data are presented as mean ± SD.(I,J) Subcellular localization of CCDC719- 13 in (I) PC3 and (J) DU145 cells. Relative RNA levels in the nucleus and cytoplasm are shown, with U6 and GAPDH as controls. **p < 0.01, ***p < 0.001.

Supplementary Figure S5. Functional Characterization and Impact of CCDC719-13 Overexpression in Prostate Cancer Cells.


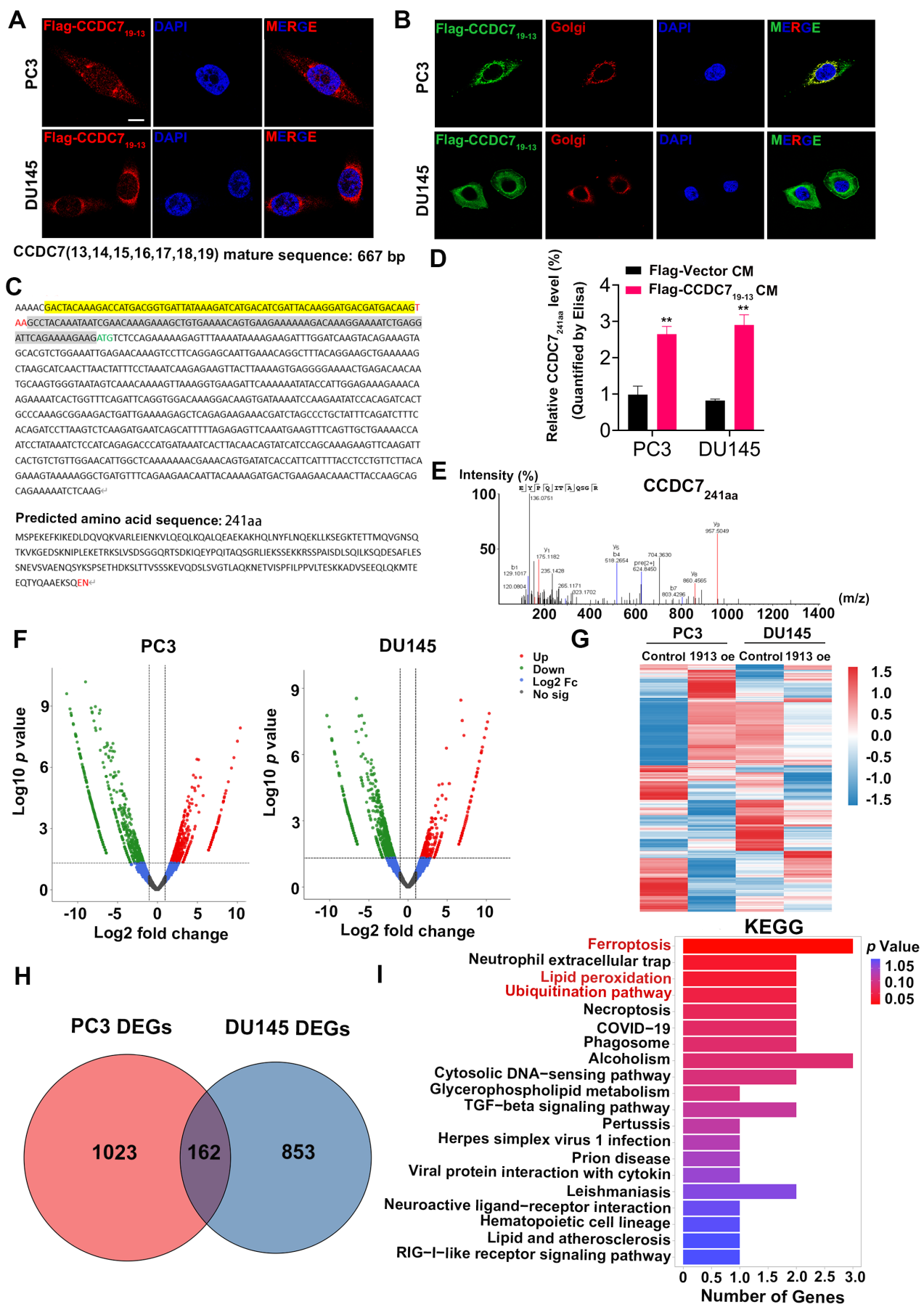


(A) Immunofluorescence analysis of Flag-tagged CCDC719-13 in PC3 and DU145 cells. Cells were stained with anti-Flag (green) and DAPI (blue) to visualize nuclei. Scale bar: 10 μ m.(B) Co-localization of Flag-tagged CCDC719-13 with Golgi apparatus marker in PC3 and DU145 cells. Cells were stained with anti-Flag (green),

Golgi marker (red), and DAPI (blue) to visualize nuclei. Scale bar: 10 μ m.(C) Nucleotide and predicted amino acid sequences of CCDC719- 13. The mature sequence spans 667 bp, encoding a CCDC7241aa protein.(D) Quantification of CCDC719-13 protein levels in PC3 and DU145 cells transfected with Flag-Vector CM or Flag-CCDC719- 13 CM, as measured by ELISA. Data are presented as mean ± SD. (E) Mass spectrometry analysis confirming the amino acid sequence of the immunoprecipitated CCDC719-13 protein. (F) Volcano plots of differentially expressed genes (DEGs) in PC3 and DU145 cells overexpressing CCDC719-13 compared to control. DEGs with log2 fold change > 1 and p-value < 0.05 are highlighted in red (upregulated) and green (downregulated).(G) Heatmap of DEGs in PC3 and DU145 cells overexpressing CCDC719-13, showing upregulated (red) and downregulated (blue) genes compared to control cells.(H) Venn diagram showing the overlap of DEGs between PC3 and DU145 cells overexpressing CCDC719-13.(I) KEGG pathway enrichment analysis of DEGs in PC3 and DU145 cells overexpressing CCDC719-13.

Supplementary Figure S6. Interaction Network and Functional Impact of CCDC719-13 in Prostate Cancer Cells.


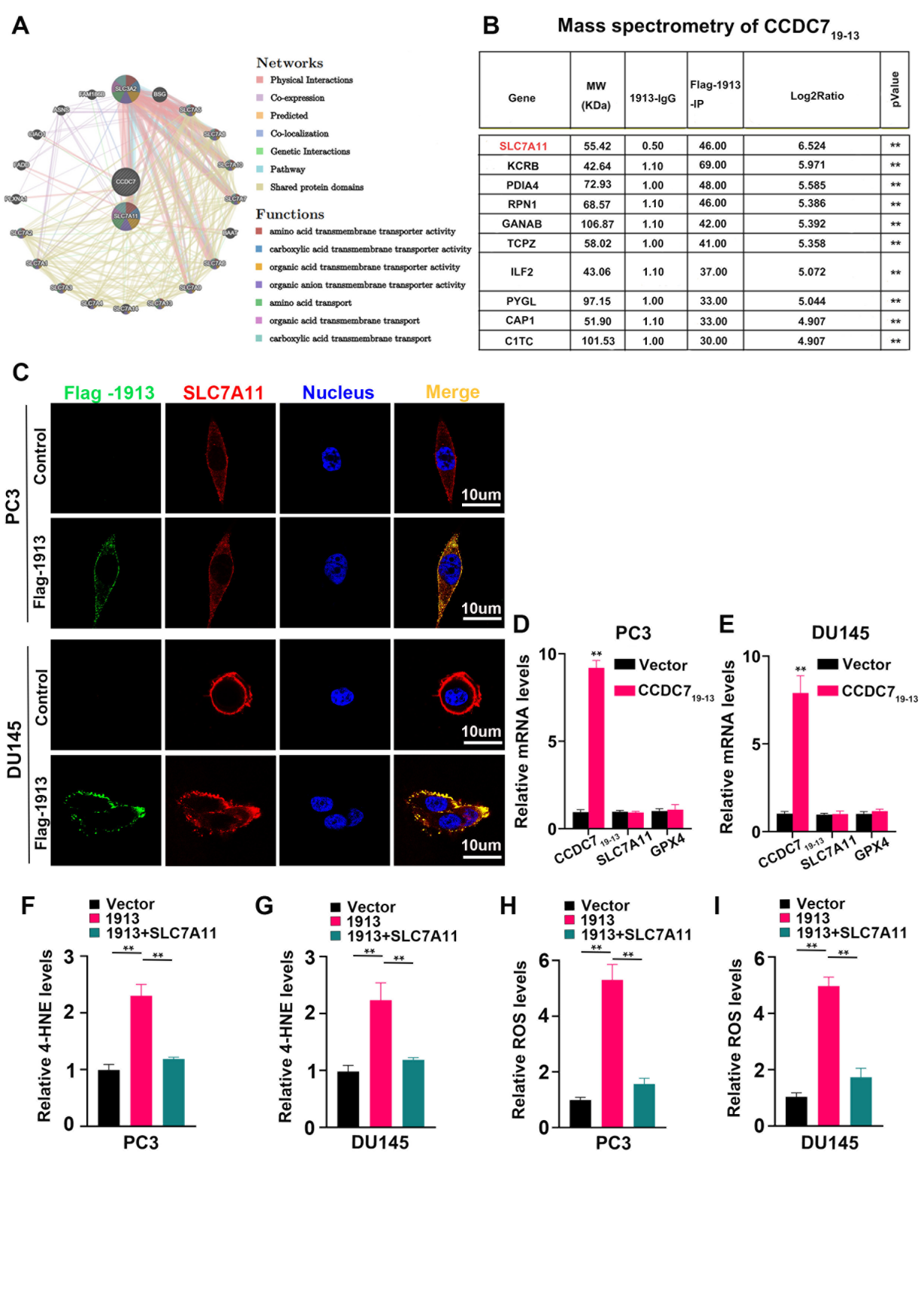


(A) Interaction network of CCDC719-13 as predicted by functional protein association networks. The network shows physical interactions, co-expression, predicted interactions, co-localizations, genetic interactions, pathways, and shared protein domains. (B) Mass spectrometry analysis identifying proteins interacting with CCDC719-13. The table lists the identified proteins, their molecular weights (MW), relative intensities, log2 ratios, and p-values. (C) Immunofluorescence analysis showing co-localization of Flag-tagged CCDC719-13 (green) with SLC7A11 (red) in PC3 and DU145 cells. PC3 and DU145 cells were transfected with vector or

Flag-CCDC719-13 plasmid and then co-cultured with wild-type PC3 and DU145 cells. Nuclei are stained with DAPI (blue). Merged images demonstrate co-localization of CCDC719-13 and SLC7A11. (D,E) Relative mRNA levels of CCDC719- 13, SLC7A11, and GPX4 in (D) PC3 and (E) DU145 cells transfected with control vector or CCDC719-13 overexpression vector, as determined by qRT-PCR. Data are presented as mean ± SD. (F-I) Relative ferroptosis levels in PC3 and DU145 cells transfected as indicated were determined by 4-HNE (F,G) and ROS (H,I) levels.

Supplementary Figure S7. Interaction and Functional Analysis of CCDC719- 13,TRIM21 and SLC7A11 in Prostate Cancer Cells.


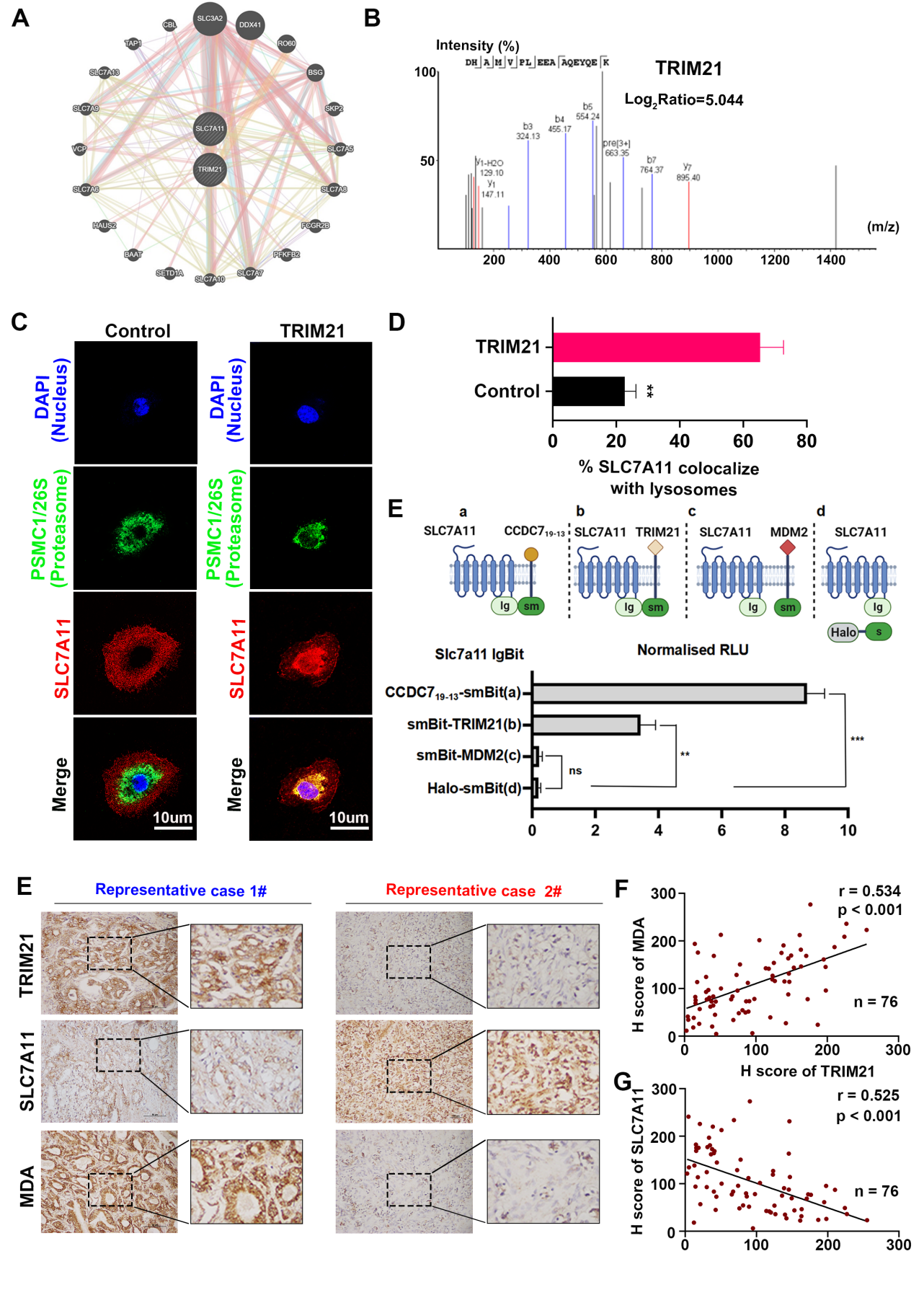


(A) Interaction network of TRIM21 and SLC7A11 as predicted by functional protein association networks. The network shows physical interactions, co-expression, predicted interactions, co-localizations, genetic interactions, pathways, and shared protein domains. Functions associated with the interacting proteins are highlighted.(B)

Mass spectrometry analysis identifying TRIM21 as an interacting protein with a significant log2 ratio of 5.044. The peptide intensity and mass-to-charge ratio (m/z) are shown.(C) Immunofluorescence analysis showing colocalization of SLC7A11 (red) with the proteasome marker PSMC1/26S (green) in control and TRIM21-overexpressing cells. Nuclei are stained with DAPI (blue). Merged images demonstrate increased colocalization of SLC7A11 with the proteasome in TRIM21-overexpressing cells. (D) Quantification of the percentage of SLC7A11 colocalizing with lysosomes in control and TRIM21-overexpressing cells. Data are presented as mean ± SD. **p < 0.01.(E) Bioluminescence resonance energy transfer (BRET) assay illustrating the interactions between SLC7A11 and CCDC719-13, TRIM21, and MDM2. Schematic representation of the assay is shown on the left, and the normalized relative light units (RLU) are shown on the right. (F, G) Representative immunohistochemistry (IHC) images showing expression levels of TRIM21, SLC7A11, and MDA in clinical prostate cancer cases. Scale bars: 100 μm. Correlation analysis between the H scores of TRIM21 and MDA (F) and between the H scores of SLC7A11 and TRIM21 (G) in prostate cancer tissues. Pearson correlation coefficients (r) and p-values are indicated.

Supplementary Figure S8. Impact of TRIM21 and CCDC7_241aa_ on Prostate Cancer Progression and Prognosis.


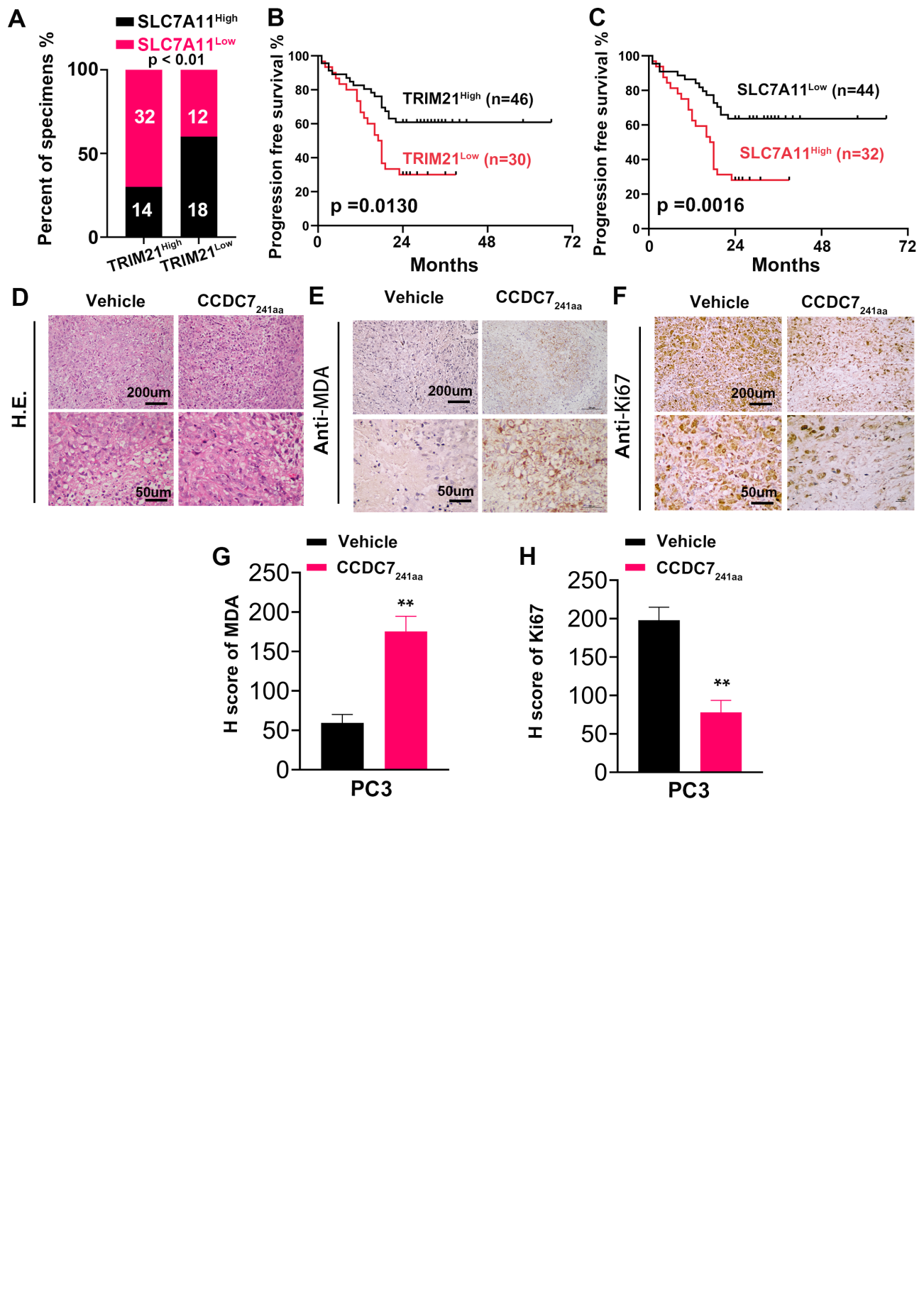


1. Proportion of prostate cancer specimens with high or low SLC7A11 expression stratified by TRIM21 expression levels.(B) Kaplan-Meier analysis of progression-free survival in prostate cancer patients with high (n=46) and low (n=30) TRIM21 expression levels. (C) Kaplan-Meier analysis of progression-free survival in prostate cancer patients s with high (n=32) and low (n=44) SLC7A11 expression levels. (D) Hematoxylin and eosin (H.E.) staining of PC3 tumor xenografts from mice treated with vehicle or CCDC7241aa. Representative images are shown at two magnifications. Scale bars: 200 μm and 50 μm.(E) Immunohistochemistry (IHC) analysis of MDA expression in PC3 tumor xenografts from mice treated with vehicle or CCDC7241aa. Representative images are shown. Scale bars: 200 μm and 50 μm.(F) IHC analysis of Ki67 expression in PC3 tumor xenografts from mice treated with vehicle or CCDC7241aa. (G) Quantification of MDA expression levels in PC3 tumor xenografts from mice treated with vehicle or CCDC7241aa. H scores are presented as mean ± SD. **p < 0.01.

Supplementary Figure S9. Safety and Efficacy of CCDC7241aa Treatment in Prostate Cancer Xenograft Models.


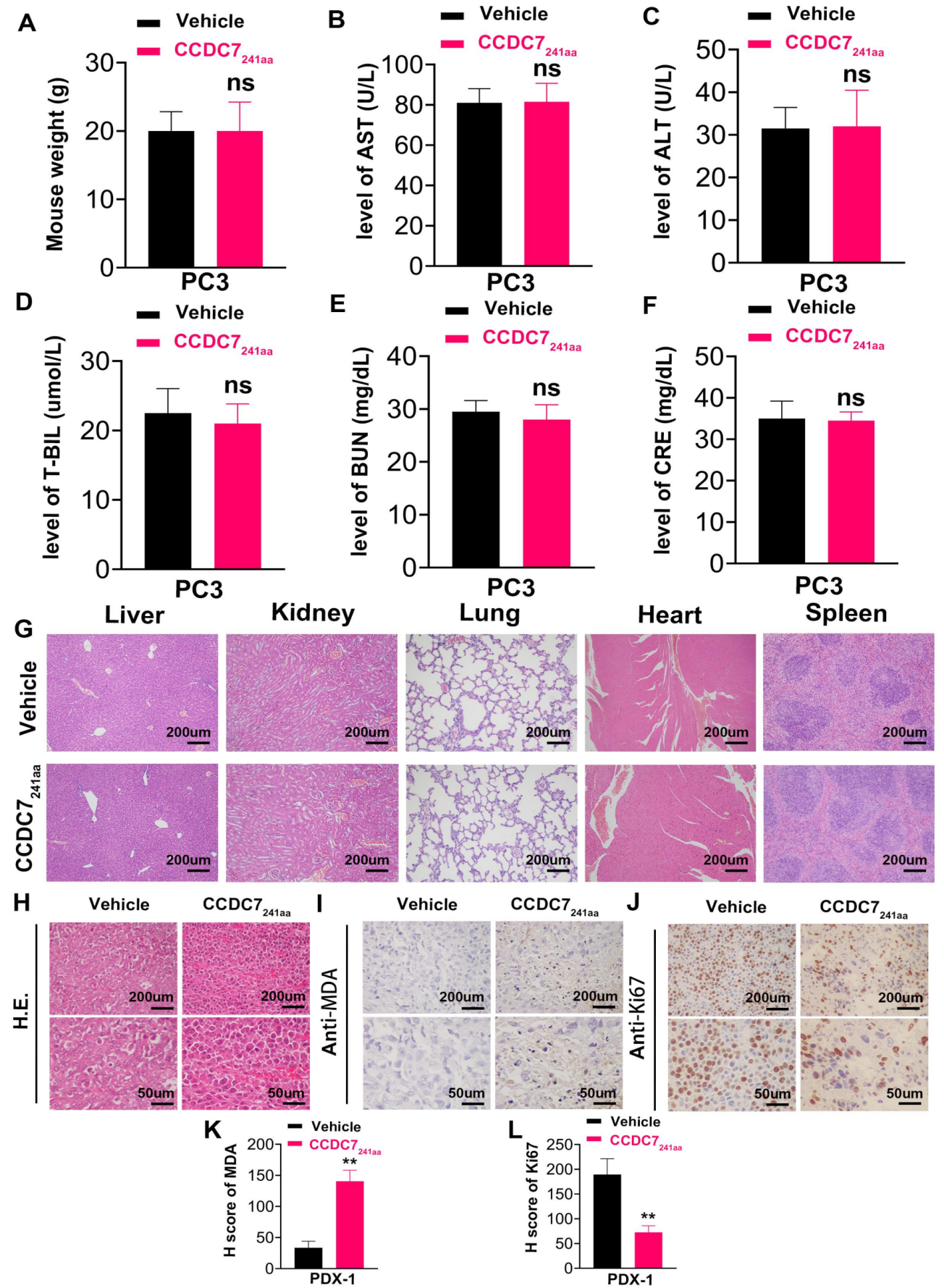


(A-F) Assessment of systemic toxicity in mice treated with vehicle or CCDC7241aa . (A) Mouse weight, (B) aspartate aminotransferase (AST) levels, (C) alanine aminotransferase (ALT) levels, (D) total bilirubin (T-BIL) levels, (E) blood urea

nitrogen (BUN) levels, and (F) creatinine (CRE) levels. Data are presented as mean ± SD. (G) Hematoxylin and eosin (H.E.) staining of major organs (liver, kidney, lung, heart, spleen) from mice treated with vehicle or CCDC7241aa. Representative images are shown. Scale bars: 200 μ m.(H) H.E. staining of PC3 tumor xenografts from mice treated with vehicle or CCDC7241aa. Representative images are shown at two magnifications. Scale bars: 200 μ m and 50 μ m.(I) Immunohistochemistry (IHC) analysis of MDA expression in PC3 tumor xenografts from mice treated with vehicle or CCDC7241aa. Representative images are shown. Scale bars: 200 μ m and 50 μ m.(J) IHC analysis of Ki67 expression in PC3 tumor xenografts from mice treated with vehicle or CCDC7241aa. Representative images are shown. Scale bars: 200 μ m and 50 μ m.(K) Quantification of MDA expression levels in PC3 tumor xenografts from mice treated with vehicle or CCDC7241aa . H scores are presented as mean ± SD. **p < 0.01.(L) Quantification of Ki67 expression levels in PC3 tumor xenografts from mice treated with vehicle or CCDC7241aa . H scores are presented as mean ± SD. **p < 0.01.

Supplementary Figure S10.


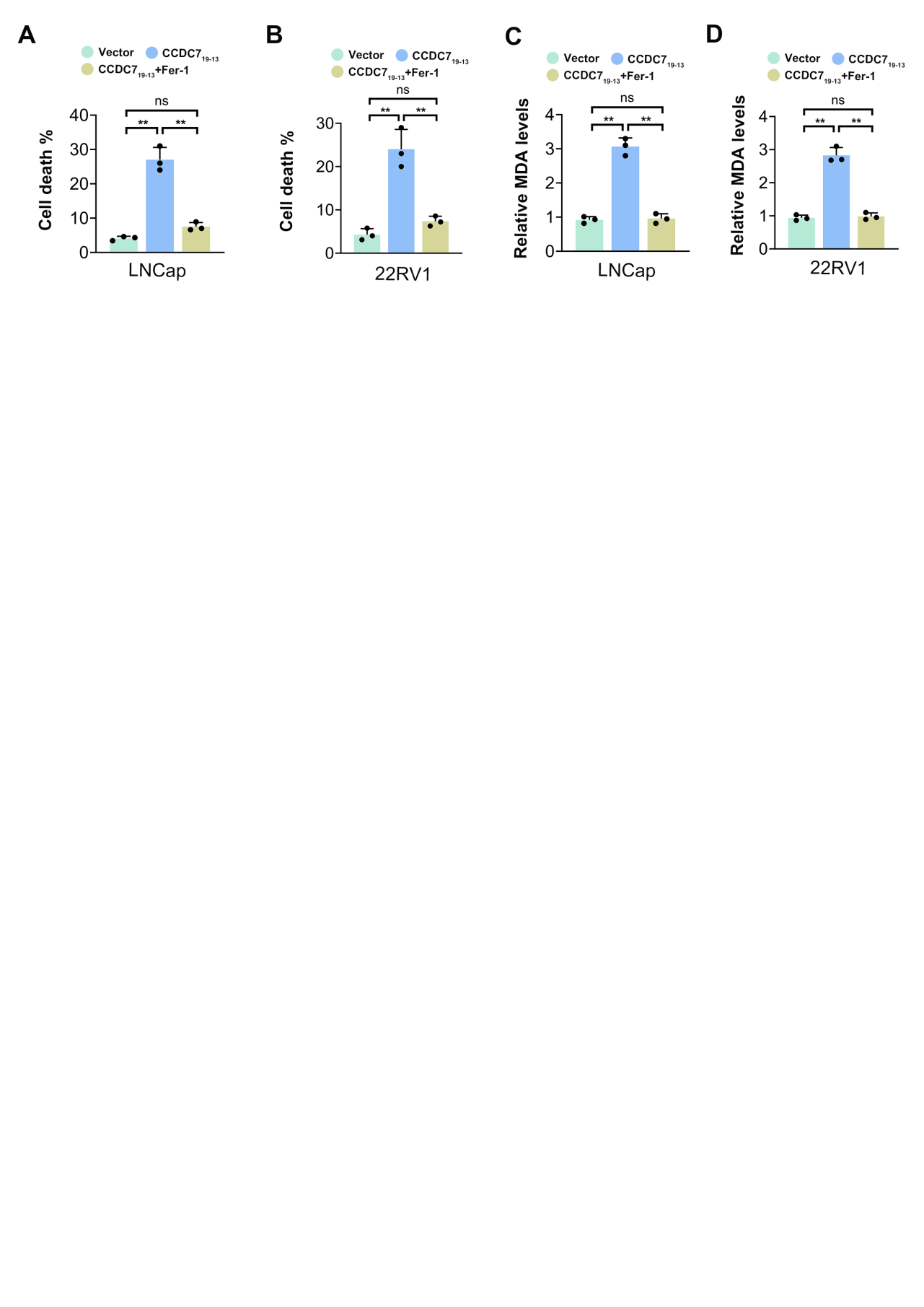


(A,B) Quantification of cell death in AR-positive prostate cancer cell lines LNCaP (A) and 22RV1 (B) transfected with vector control, CCDC7_19-13_, or CCDC7_19-13_ plus ferroptosis inhibitor ferrostatin-1 (Fer-1), as indicated. Cell death was measured by flow cytometry and presented as percentage.(C,D) Relative ferroptosis levels in LNCaP (C) and 22RV1 (D) cells transfected as indicated were assessed by malondialdehyde (MDA) quantification. CCDC7_19-13_ expression significantly increased both cell death and MDA levels, which were reversed by Fer-1 treatment. ns, not significant; **, P < 0.01.
